# The Small RNA RyhB Is a Regulator of Cytochrome Expression in *Shewanella oneidensis*

**DOI:** 10.3389/fmicb.2018.00268

**Published:** 2018-02-21

**Authors:** Karin L. Meibom, Elena M. Cabello, Rizlan Bernier-Latmani

**Affiliations:** ^1^Environmental Microbiology Laboratory, School of Architecture, Civil and Environmental Engineering, École Polytechnique Fédérale de Lausanne, Lausanne, Switzerland; ^2^Bioinformatics and Biostatistics Core Facility, École Polytechnique Fédérale de Lausanne, Lausanne, Switzerland

**Keywords:** *Shewanella oneidensis*, cytochromes, RyhB, sRNA, Fur

## Abstract

*Shewanella oneidensis* produces an extensive electron transfer network that results in metabolic flexibility. A large number of *c*-type cytochromes are expressed by *S. oneidensis* and these function as the fundamental electron transport chain proteins. Although several *S. oneidensis* cytochromes have been well-characterized, little is known about how their expression is regulated. In this study, we investigate the role of the ferric uptake regulator (Fur) and the sRNA RyhB in regulation. Our results demonstrate that loss of Fur leads to diminished growth and an apparent decrease in heme-containing proteins. Remarkably, deleting the Fur-repressed *ryhB* gene almost completely reverses these physiological changes, indicating that the phenotypes resulting from loss of Fur are (at least partially) dependent on RyhB. RNA sequencing identified a number of possible RyhB repressed genes. A large fraction of these encode *c*-type cytochromes, among them two of the most abundant periplasmic cytochromes CctA (also known as STC) and ScyA. We show that RyhB destabilizes the mRNA of four of its target genes, *cctA, scyA, omp35*, and *nrfA* and this requires the presence of the RNA chaperone Hfq. Iron limitation decreases the expression of the RyhB target genes *cctA* and *scyA* and this regulation relies on the presence of both Fur and RyhB. Overall, this study suggests that controlling cytochrome expression is of importance to maintain iron homeostasis and that sRNAs molecules are important players in the regulation of fundamental processes in *S. oneidensis* MR-1.

## Introduction

The gammaproteobacterium *Shewanella oneidensis* is a facultative anaerobe that is remarkable for its metabolic versatility. In the absence of dissolved oxygen, it can utilize a large number of soluble compounds as terminal electron acceptors, and is furthermore capable of transferring electrons to reduce insoluble metal oxides in the extracellular environment (for review, see Hau and Gralnick, 2007). This remarkable respiratory capacity has been attributed to the large number of *c*-type cytochromes encoded in the genome of *S. oneidensis* MR-1 and serving as fundamental electron transport chain proteins (Meyer et al., 2004). A total of 42 possible *c*-type cytochrome genes were identified, but one of these is a pseudogene (Meyer et al., 2004). Some, but not all, of the remaining 41 *c*-type cytochromes have been characterized and their function determined, with most scrutiny given to the proteins involved in electron transfer to ferric iron.

Reduction of insoluble iron compounds occurs at the bacterial surface and requires electron transfer from the quinone pool in the cytoplasmic membrane to the terminal reductases located in the outer membrane. CymA is a tetraheme cytochrome attached to the periplasmic side of the cytoplasmic membrane. It delivers electrons to multiple partners in the periplasmic space and is required for the reduction of Fe(III), DMSO, fumarate, nitrate, and nitrite (Myers and Myers, 1997, 2000; Schwalb et al., 2003). The most abundant periplasmic *c*-type cytochromes are the small tetraheme CctA (also called STC), the fumarate reductase FccA, and the small monoheme ScyA (Tsapin et al., 2001; Meyer et al., 2004), all of which have been shown to be involved in direct electron transfer with CymA (Schwalb et al., 2003; Schuetz et al., 2009; Firer-Sherwood et al., 2011; Schütz et al., 2011; Fonseca et al., 2013). In addition to CymA, reduction of Fe(III) oxides by *S. oneidensis* relies on the proteins MtrA, MtrB, MtrC, and OmcA (for review, see Richter et al., 2012; Shi et al., 2012). MtrA is a periplasmic decaheme *c*-type cytochrome that also associates with the outer membrane β-barrel protein MtrB and the outer membrane decaheme *c*-type cytochrome MtrC. OmcA is another decaheme *c*-type cytochrome associated with the outer surface that interacts with MtrC. MtrA reportedly transfers electrons across the outer membrane to the terminal reductases, MtrC and OmcA, through the porin formed by MtrB. MtrA can accept electrons directly from CymA or presumably indirectly via other periplasmic cytochromes, such as CctA and FccA (Schwalb et al., 2003; Schuetz et al., 2009; Firer-Sherwood et al., 2011; Fonseca et al., 2013; Sturm et al., 2015).

The presence of individual anaerobic electron acceptors seems, for the most part, not to regulate expression of specific *c*-type cytochromes (Beliaev et al., 2005) (with the exception of the nitrite reductase NrfA), and a core set of periplasmic cytochromes are expressed regardless of electron acceptor (Sturm et al., 2015). Instead, a number of *c*-type cytochromes respond to oxygen availability (Jin et al., 2013; Barchinger et al., 2016), and iron availability (Yang et al., 2009).

Iron is an essential nutrient for most bacteria as a protein cofactor in pathways such as respiration, metabolism and genome repair (for review, see Andrews et al., 2003; Frawley and Fang, 2014). It is the most common redox active metal in proteins and is found in heme and iron-sulfur groups. To ensure sufficient intracellular iron for protein cofactors, bacteria employ a number of iron scavenging and iron uptake systems. However, maintaining iron homeostasis is of crucial importance because free iron is potentially damaging to the bacterial cell due to radicals formed by Fenton chemistry. As a result, iron uptake and iron storage are tightly regulated. Many bacteria use the transcription factor Fur (ferric uptake regulator) as the major regulator of iron homeostasis. Fur binds Fe^2+^ and typically acts directly as a repressor of transcription, by binding to DNA recognition sequences when the iron concentration is sufficiently high. In *S. oneidensis*, deletion of *fur* results in a pale color of the colonies formed by the Δ*fur* strain compared to the reddish color of the wild-type *S. oneidensis* colonies, suggesting less heme is present in the Δ*fur* strain (Yang et al., 2008).

Under iron limiting conditions, Fur-dependent repression is relieved and Fur-repressed genes are expressed, including the small RNA RyhB in *E. coli* and other species (Massé and Gottesman, 2002; Oglesby-Sherrouse and Murphy, 2013). In the studied bacterial genera (*Escherichia* and *Vibrio*), one of the functions of Fur and RyhB is to control global iron usage (Massé et al., 2005; Mey et al., 2005).

Small RNAs (sRNAs) are regulators of many cellular processes and play important roles in adaptive changes. They are frequently transcribed after exposure to stress and act as post-transcriptional regulators. They generally act by base pairing *in trans* with sequences in target mRNAs, either blocking or freeing access to the ribosome binding site. Base pairing between a sRNA and mRNA commonly affect mRNA stability by promoting degradation by RNases or alternatively preventing RNase cleavage (Nitzan et al., 2017). RyhB has been extensively studied in *E. coli* where it most commonly negatively regulates genes by base-pairing with their mRNA and affecting translation and/or transcript stability, but where it can also affect expression positively (Oglesby-Sherrouse and Murphy, 2013).

Here, we present a study of the role of Fur and RyhB in *S. oneidensis* physiology and evidence their impact on growth and production of heme-containing proteins. We identify a number of genes as potential targets of RyhB regulation and demonstrate that RyhB affects the stability of specific *c*-type cytochrome mRNA transcripts. The findings imply that iron levels control production of cytochrome expression in *S. oneidensis* through Fur and RyhB.

## Materials and methods

### Bacterial strains and culture conditions

Bacterial strains and plasmids used in this study are listed in Supplementary Table 1. *S. oneidensis* MR-1 wild-type and mutants were routinely grown in LB medium at 30°C and supplemented with 5 μg ml^−1^ tetracycline and/or 50 μg ml^−1^ kanamycin when indicated. All experiments were performed with oxygen as the electron acceptor and bacterial cultures were grown in Erlenmeyer flasks with shaking.

*E. coli* strains DH5α or DH5αλ*pir*, used for cloning purposes, were grown in LB medium at 37°C supplemented with 5 μg ml^−1^ tetracycline or 50 μg ml^−1^ kanamycin when indicated. Diaminopilimic acid (DAP) was added to LB medium for growth of strain WM3064, which was used for conjugation of plasmids into *S. oneidensis*. The DAP dependence of WM3064 was utilized as counter-selection tool after conjugation.

### Construction of Δ*fur*, Δ*ryhB*, Δ*fur*Δ*ryhB*, and Δ*hfq* mutants of *S. oneidensis* MR-1

The yeast recombineering vector pMQ150 (Shanks et al., 2009) was digested with *Nhe*I and *Not*I, treated with Klenow enzyme and ligated. The resulting suicide vector, pMQS, was used as a backbone plasmid for targeted gene deletions in *S. oneidensis* MR-1. Plasmids for construction of markerless gene deletions were constructed by amplifying regions of 500 to 600 bp flanking the individual genes (using primers D*gene*_5F+D*gene*_5R and D*gene*_3F+D*gene*_3R, respectively). All primers used in this study are listed in Supplementary Table 2. Deletions in *fur* and *hfq* were in-frame and left the sequences encoding the first four amino acids and the last four or two amino acids, respectively, of the two proteins. Crossover PCR was performed to join the up- and down-stream regions followed by cloning into pMQS. pMQS-derivatives containing inserts for deletion of *fur, ryhB*, or *hfq* were introduced in *S. oneidensis* MR-1 by conjugation from *E. coli* strain WM3064. Colonies with single crossover plasmid insertions were selected on LB agar plates containing kanamycin, purified once on agar plates with kanamycin and resistant colonies were subsequently grown overnight in LB (containing no NaCl) without antibiotic. Double crossover mutants were selected on LB agar plates (containing no NaCl) supplemented with 10% sucrose. Sucrose resistant and kanamycin sensitive colonies were checked by colony PCR for gene deletion using primers flanking the deleted region (*gene*_FO + *gene*_RO). Selected clones were purified, genomic DNA isolated, and the region containing the deleted gene was amplified by PCR and the deletion verified by Sanger sequencing. The double mutant, Δ*fur*Δ*ryhB*, was made by deleting *ryhB* in the Δ*fur* mutant.

### Construction of pKM033 and pKM033-ryhB

Plasmid pKM001 was constructed by digesting pBAD-fccA-His (Schuetz et al., 2009) with *Eco*RV and ligating the vector backbone, thereby deleting almost the entire *fccA* gene and part of *araC*. The *E. coli lacI* gene was amplified by PCR (using primers ELacI_F+ELacI_R) from plasmid pET28b(+) and cloned into the *Eco*RV site of pKM001 after digestion with *Sma*I. An artificial promoter containing a Lac operator between the −35 and −10 motifs was created by annealing two oligonucleotides (PlacO_f and PlacO_r), followed by insertion into the *Hind*III site, thereby creating pKM033. The sequence from −1 to −6 relative to transcription start in this construct is a *Bam*HI restriction site, allowing cloning of genes resulting in transcription initiating at their native transcription start site. In addition, a *Kpn*I site is located at position +1 to +6. The *ryhB* gene was amplified by PCR (using primers ryhB_F_(+1) and ryhB_R_Stop) and the PCR product inserted into *Bam*HI/*Hind*III digested pKM033 after digestion with the same enzymes. The transcription start site of *ryhB* (from Shao et al., 2014) follows the *Bam*HI site at position +1 in plasmid KM033.

### RNA sequencing

Triplicate cultures of *S. oneidensis* MR-1 and mutants were independently grown in LB medium to OD_600nm_ ~0.7. For Δ*ryhB* containing either pKM033 or pKM033-ryhB, triplicate independent cultures were grown in LB medium to OD_600nm_ ~0.2 at which point 0.5 mM IPTG was added and growth continued for 60 min. Cells were harvested and total RNA isolated using Trizol reagent (Invitrogen) followed by purification of the aqueous phase on a RNeasy column (Qiagen). RNA was treated with DNase I for 45 min at 37°C and again purified on an RNeasy column. The integrity of the RNA was assessed on a fragment analyzer (Advanced Analytical Technologies, Ankeny, Iowa, USA). RNA sequencing libraries were constructed after rRNA depletion (with the Illumina Ribo-Zero rRNA removal kit) using the Truseq stranded mRNA library prep kit (without polyA selection using the strand-specific kit (Illumina, San Diego, CA, USA)). Library construction and RNA sequencing was performed at the Lausanne Genomic Technologies Facility at the University of Lausanne, Switzerland.

### Analysis of RNAseq data

RNA sequencing reads were aligned with TopHat2 v.2.0.11 (Kim et al., 2013) (parameters: –library-type fr-firststrand -p 6 -g 2 -G). Reads were counted using featureCounts (Liao et al., 2014) (parameters: -t CDS -g gene_name -s 2), included in the SourceForge Subread v.1.5.1 package (Liao et al., 2013). Reference genome and annotation files were downloaded from NCBI RefSeq database (NCBI reference: chromosome: NC_004347.2; megaplasmid: NC_004349.1.).

Once the matrix of read counts was created, only those genes having more than 10 counts per million mapped reads (CPM) in at least 3 of all the samples were kept. Normalization and conversion to logCPM, was performed using the edgeR package v.3.14.0 (Robinson et al., 2010), and the limma-trend approach from the Limma package (Ritchie et al., 2015), in order to complete the differential expression analysis. Both edgeR and Limma are R Bioconductor packages (Huber et al., 2015).

Only genes which adj.p.value < 0.05 and logFC > |1| were considered differentially expressed. Genes expressed at lower level in Δ*fur* relative to wild-type according to these criteria are shown in Table 1.

**Table 1:** RNAseq data of genes expressed at lower level in Δ*fur* mutant relative to wild-type *S. oneidensis* MR-1^a^.

		**Δ*****fur*** **vs. WT**		**Δ*****fur***Δ***ryhB*** **vs**. Δ***fur***		**Δ*****fur***Δ***ryhB*** **vs. WT**		**RyhB overexpression^b^**		
**Locus**	**Annotation**	**log_2_R**	**adj.P.Val**	**log_2_R**	**adj.P.Val**	**log_2_R**	**adj.P.Val**	**log_2_R**	**adj.P.Val**	**Group^c^**
SO_0002	Glutathione uptake transporter	**−1.02**	**4.9E-06**	**0.80**	**1.39E-04**	−0.22	2.41E-01	**−0.67**	**7.17E-04**	Ia
SO_0264	Periplasmic monoheme cytochrome c5 ScyA	**−2.76**	**1.7E-10**	**2.71**	**2.16E-10**	−0.04	8.18E-01	**−3.57**	**5.32E-12**	Ia
SO_0608	Ubiquinol-cytochrome c reductase iron-sulfur subunit Peta	**−1.04**	**4.0E-07**	**0.91**	**5.55E-06**	−0.12	4.21E-01	**−1.29**	**1.26E-07**	Ia
SO_0609	Ubiquinol-cytochrome c reductase cytochrome b subunit Petb	**−1.11**	**1.9E-07**	**0.99**	**2.46E-06**	−0.12	4.11E-01	**−1.40**	**5.47E-08**	Ia
SO_0610	Ubiquinol-cytochrome c reductase cytochrome c1 subunit Petc	**−1.01**	**3.0E-08**	**0.88**	**6.01E-07**	−0.13	2.44E-01	**−1.23**	**1.30E-08**	Ia
SO_1236	Purine transporter	**−1.23**	**4.7E-06**	**1.08**	**6.43E-05**	−0.16	5.09E-01	**−0.97**	**1.67E-04**	Ia
SO_1718	Protein of unknown function	**−1.18**	**8.2E-05**	**0.92**	**1.57E-03**	−0.26	3.86E-01	**−1.09**	**6.65E-04**	Ia
SO_2373	Drug:H+ antiporter	**−1.17**	**3.3E-06**	**0.91**	**1.18E-04**	−0.26	2.01E-01	**−0.80**	**3.74E-04**	Ia
SO_2727	Periplasmic tetraheme cytochrome c CctA	**−1.97**	**1.1E-08**	**1.88**	**8.31E-08**	−0.09	6.94E-01	**−2.19**	**1.30E-08**	Ia
SO_2797	Glutaredoxin	**−1.05**	**1.3E-03**	**0.71**	**2.46E-02**	−0.34	3.68E-01	**−0.75**	**3.04E-02**	Ia
SO_2881	Superoxide dismutase SodB	**−1.42**	**1.1E-07**	**1.27**	**1.69E-06**	−0.15	4.09E-01	**−2.22**	**5.82E-09**	Ia
SO_3195	Proton-dependent oligopeptide transporter	**−1.07**	**5.6E-07**	**0.93**	**9.75E-06**	−0.15	3.50E-01	**−1.16**	**8.58E-07**	Ia
SO_3420	Monoheme cytochrome c'	**−1.22**	**1.2E-06**	**1.26**	**3.48E-06**	0.04	8.63E-01	**−2.09**	**1.34E-08**	Ia
SO_3920	Periplasmic [Fe-Fe] hydrogenase large subunit HydA	**−1.79**	**2.7E-06**	**1.46**	**6.89E-05**	−0.33	2.77E-01	**−0.87**	**4.52E-03**	Ia
SO_3980	Ammonia-forming nitrite reductase NrfA	**−1.95**	**1.6E-05**	**1.43**	**7.04E-04**	−0.53	1.98E-01	**−1.53**	**5.69E-04**	Ia
SO_4047	SoxA-like diheme cytochrome c	**−1.11**	**6.5E-06**	**1.51**	**1.59E-06**	0.40	5.19E-02	**−1.63**	**5.14E-07**	Ia
SO_4340	Putative transport protein	**−1.47**	**5.9E-06**	**0.96**	**6.61E-04**	−0.50	6.09E-02	**−0.59**	**2.19E-02**	Ia
SO_4506	Iron-sulfur cluster-binding protein	**−1.20**	**2.1E-05**	**1.11**	**1.51E-04**	−0.10	7.50E-01	**−0.67**	**8.00E-03**	Ia
SO_4507	Formate dehydrogenase chaperone FdhT	**−1.08**	**1.1E-05**	**0.96**	**1.17E-04**	−0.12	6.01E-01	**−0.83**	**4.71E-04**	Ia
SO_4509	Formate dehydrogenase molybdopterin-binding subunit FdhA	**−1.30**	**3.4E-05**	**1.17**	**2.90E-04**	−0.13	6.86E-01	**−0.68**	**1.54E-02**	Ia
SO_4510	Formate dehydrogenase FeS subunit FdhB	**−1.05**	**2.6E-06**	**0.97**	**2.27E-05**	−0.08	6.75E-01	**−0.70**	**3.74E-04**	Ia
SO_4591	Membrane anchored tetraheme cytochrome c CymA	**−1.75**	**2.3E-05**	**1.40**	**5.00E-04**	−0.35	3.68E-01	**−0.90**	**1.32E-02**	Ia
SO_0403	Predicted outer membrane protein	**−1.39**	**2.5E-03**	**1.50**	**3.36E-03**	0.11	8.77E-01	−0.61	2.30E-01	Ib
SO_0404	Zinc dependent metalloprotease domain lipoprotein	**−1.33**	**5.6E-05**	**1.51**	**7.44E-05**	0.18	6.07E-01	−0.54	6.49E-02	Ib
SO_0435	Uroporphyrinogen decarboxylase HemE	**−1.20**	**4.0E-05**	**0.92**	**1.04E-03**	−0.28	3.15E-01	−0.27	3.00E-01	Ib
SO_0827	Lactate permease	**−1.81**	**8.9E-08**	**1.51**	**2.43E-06**	−0.30	1.68E-01	−0.34	9.19E-02	Ib
SO_1072	N-acetylglucosamine-binding protein A GbpA	**−2.24**	**3.1E-06**	**1.77**	**9.80E-05**	−0.47	2.30E-01	−0.25	5.53E-01	Ib
SO_1192	Predicted extracytoplasmic protein	**−1.11**	**1.6E-04**	**1.06**	**7.01E-04**	−0.06	8.81E-01	0.09	8.09E-01	Ib
SO_2806	Phospahte starvation inducible E-like protein	**−1.27**	**9.3E-06**	**0.81**	**1.12E-03**	−0.46	5.93E-02	−0.30	1.98E-01	Ib
SO_2824	Pseudogene	**−2.92**	**5.9E-04**	**2.86**	**1.66E-03**	−0.06	9.57E-01	1.46	8.81E-02	Ib
SO_2905	O-methyltransferase	**−1.23**	**3.4E-04**	**0.86**	**8.67E-03**	−0.37	3.20E-01	−0.35	3.26E-01	Ib
SO_3284	Protein YbgT	**−1.24**	**4.2E-05**	**0.79**	**3.57E-03**	−0.45	1.20E-01	−0.19	5.38E-01	Ib
SO_3285	Cytochrome d ubiquinol oxidase subunit II CydB	**−1.24**	**3.6E-05**	**0.80**	**2.83E-03**	−0.43	1.24E-01	−0.20	4.82E-01	Ib
SO_3286	Cytochrome d ubiquinol oxidase subunit I CydA	**−1.27**	**7.0E-05**	**0.90**	**2.61E-03**	−0.37	2.38E-01	−0.21	5.16E-01	Ib
SO_3705	5-Methylthioadenosine nucleosidase/S-adenosylhomocysteine nucleosidase	**−1.79**	**5.6E-07**	**1.39**	**2.45E-05**	−0.40	1.23E-01	0.09	7.67E-01	Ib
SO_3706	Nucleoside:proton symporter NupX	**−1.90**	**2.8E-07**	**1.50**	**1.16E-05**	−0.41	1.11E-01	0.23	3.74E-01	Ib
SO_3874	Transcriptional regulator LysR family	**−1.17**	**4.7E-04**	**0.67**	**2.92E-02**	−0.50	1.68E-01	−0.09	8.57E-01	Ib
SO_4085	Chitinase ChiA	**−1.05**	**5.6E-05**	**0.66**	**4.97E-03**	−0.40	1.16E-01	−0.30	2.13E-01	Ib
SO_4138	Putative periplasmic protein	**−1.07**	**1.8E-04**	**0.94**	**1.31E-03**	−0.13	7.05E-01	−0.06	8.86E-01	Ib
SO_4157	Thiosulfate/tetrathionate-responsive two component signal transduction system response regulator Ttr	**−1.00**	**2.0E-03**	**1.07**	**3.03E-03**	0.06	8.96E-01	−0.14	7.66E-01	Ib
SO_4274	Undecaprenol diphosphatase UppP	**−1.49**	**3.8E-07**	**1.18**	**1.49E-05**	−0.31	1.32E-01	0.24	2.17E-01	Ib
SO_4513	Fnr-inducilble formate dehydrogenase molybdopterin-binding subunit FdhA	**−1.07**	**2.6E-03**	**1.00**	**8.22E-03**	−0.07	8.95E-01	−0.33	4.33E-01	Ib
SO_4623	Two component signal transduction system response regulator	**−1.15**	**2.6E-03**	**1.00**	**1.15E-02**	−0.14	7.89E-01	−0.11	8.58E-01	Ib
SO_4625	Predicted phosphoribosyltransferase ComF family	**−1.33**	**1.1E-04**	**1.55**	**1.16E-04**	0.22	5.57E-01	0.02	9.72E-01	Ib
SO_4719	ABC-type tungstate uptake system substrate-binding component TupA	**−1.38**	**1.8E-05**	**0.87**	**1.99E-03**	−0.51	7.74E-02	0.01	9.81E-01	Ib
SO_4720	ABC-type tungstate uptake system permease component TupB	**−1.11**	**2.5E-05**	**0.87**	**6.39E-04**	−0.24	3.21E-01	−0.10	7.30E-01	Ib
SO_1777	Extracelllular iron oxide respiratory system periplasmic decaheme cytochrome c component MtrA	**−2.02**	**1.3E-06**	**0.59**	**3.02E-02**	**−1.43**	**1.05E-04**	**−0.91**	**4.47E-03**	IIa
SO_1778	Extracellular iron oxide respiratory system surface decaheme cytochrome c component MtrC	**−2.24**	**1.2E-06**	**0.66**	**2.87E-02**	**−1.58**	**1.05E-04**	**−0.74**	**2.64E-02**	IIa
SO_1948	Glutamate/aspartate:proton symporter GltP	**−1.13**	**1.6E-07**	**0.57**	**3.08E-04**	**−0.56**	**4.37E-04**	**−0.81**	**1.19E-05**	IIa
SO_2879	N-acetylglucosamine/uracil transporter UraA	**−1.20**	**1.6E-08**	**0.78**	**5.50E-06**	**−0.42**	**1.35E-03**	**−0.36**	**4.47E-03**	IIa
SO_3099	Outer membrane long-chain fatty acid receptor FadL family	**−1.25**	**2.0E-05**	**0.73**	**3.71E-03**	**−0.52**	**4.89E-02**	**−0.50**	**4.20E-02**	IIa
SO_3896	outer membrane porin Omp35	**−1.80**	**7.3E-08**	**1.23**	**1.30E-05**	**−0.57**	**8.77E-03**	**−1.95**	**9.64E-08**	IIa
SO_4232	Long-chain fatty acid transport protein	**−1.43**	**5.7E-07**	**0.71**	**8.58E-04**	**−0.72**	**1.24E-03**	**−1.37**	**2.56E-06**	IIa
SO_4666	Diheme cytochrome c4 CytcB	**−1.30**	**6.0E-07**	**0.91**	**6.89E-05**	**−0.39**	**3.99E-02**	**−1.36**	**1.29E-06**	IIa
SO_0141	Nitrate/nitrite-responsive bifunctional diguanylate cyclase/phosphodiesterase with PAS sensory domain	**−1.58**	**9.3E-06**	**0.96**	**1.52E-03**	**−0.62**	**4.28E-02**	−0.26	4.00E-01	IIb
SO_2384	Site-specific recombinase phage integrase family	**−1.95**	**8.7E-07**	**1.27**	**1.49E-04**	**−0.68**	**2.24E-02**	−0.03	9.45E-01	IIb
SO_4131	Nucleoside-specifc outer membrane porin Tsx family	**−1.95**	**5.9E-06**	**1.03**	**2.81E-03**	**−0.92**	**1.25E-02**	−0.25	5.01E-01	IIb
SO_4355	cAMP-binding regulator	**−1.37**	**1.6E-05**	**0.73**	**5.57E-03**	**−0.64**	**2.46E-02**	−0.21	4.77E-01	IIb
SO_1776	Extracellular iron oxide respiratory system outer membrane component MtrB	**−1.61**	**1.2E-06**	0.40	5.91E-02	**−1.21**	**5.10E-05**	**−0.73**	**4.22E-03**	III (IIa)
SO_1111	Bacterioferritin subunit 2 Bfr2	**−2.25**	**3.0E-07**	−0.16	5.42E-01	**−2.42**	**2.46E-07**	**1.29**	**2.19E-04**	III
SO_1112	Bacterioferritin subunit 1 Bfr1	**−2.22**	**3.8E-07**	−0.18	5.05E-01	**−2.40**	**2.68E-07**	**1.31**	**2.02E-04**	III
SO_1779	Extracelllular iron oxide respiratory system surface decaheme cytochrome c component OmcA	**−2.49**	**1.3E-07**	0.51	5.31E-02	**−1.98**	**2.51E-06**	0.15	6.80E-01	III
SO_1977	Inner membrane of unknown function	**−1.25**	**1.3E-05**	0.18	3.91E-01	**−1.06**	**1.92E-04**	**0.49**	**3.77E-02**	III
SO_0396	Quinol:fumarate reductase menaquinol-oxidizing subunit FrdC	**−1.38**	**1.2E-02**	0.92	1.11E-01	−0.46	5.26E-01	−0.09	9.35E-01	other

a *All genes that are downregulated at least 2-fold (and with adjusted p < 0.05) in Δfur relative to the wild-type strain are listed in the table. For these genes, expression ratios and adjusted p-values are shown for other strains comparisons. For all strain comparisons, the Log_2_R (log_2_ of the expression ratio) with adjusted p < 0.05 are indicated in bold, also when expression ratios are <2-fold*.

b *RyhB overexpression data are from strains ΔryhB/pKM033-ryhB vs. ΔryhB/pKM033*.

c *The genes were divided into groups based on their expression pattern in various strains. Group I genes are significantly (p < 0.05) upregulated in ΔfurΔryhB relative to Δfur and show no significant difference in expression between wild-type and ΔfurΔryhB. Group II genes are differently expressed in ΔfurΔryhB relative to both Δfur (upregulated) and relative to wild-type (downregulated). Group III genes are not differently expressed in ΔfurΔryhB relative to Δfur. Genes in groups Ia and IIa are downregulated after RyhB over-expression*.

The computations were performed at the Vital-IT (http://www.vital-it.ch) Center for high-performance computing of the SIB Swiss Institute of Bioinformatics.

RNAseq data are available GEO (Gene Expression Omnibus) with accession number GSE104952.

### RNA stability assay

*S. oneidensis* wild-type and mutants (with or without pKM033 or pKM033-ryhB) were grown to mid-exponential phase and transcription was stopped by adding 500 μg ml^−1^ rifampicin. For experiments with RyhB overexpression, 0.5 mM IPTG was added to the cultures for 30 min before addition of rifampicin. Samples were taken at 0 min (immediately before adding rifampicin), 4 min, 8 min, and 15 min after transcription was terminated. Cultures (2 ml) were centrifuged at maximum speed at room temperature for one min and the bacterial pellet was immediately frozen in dry ice/ethanol.

### Iron depletion experiment

Bacterial cultures were grown to mid-exponential phase in LB medium at which time iron chelator (2,2′-dipyridyl) was added at a concentration of 250 μg ml^−1^, and incubation was continued for 30 min. Samples were taken just before addition of iron chelator (Fe-rich) and at the end of the experiment (Fe-limitation).

### qRT-PCR

RNA was isolated from liquid cultures of *S. oneidensis* wild-type and mutants using Trizol reagent followed by purification of the aqueous phase on RNeasy columns (Qiagen), with either on-column or in-solution DNase digestion (in latter case followed by a second RNeasy purification step). cDNA synthesis was performed according to the protocol provided by with the reverse transcriptase GoScript (Promega) using 250–500 ng total RNA and random hexamers. qPCR of diluted cDNA was performed in 10 μl volume [containing 1xSensiFast SYBR Mix (BioLine) and 200 nM primers] in a Mic qPCR Cycler (Bio Molecular Systems) with a 2-step program (5 min 95°C, followed by 40 cycles of 95°C for 5 s and 60°C for 20 s). Four repetitions of each sample and each primer set were performed, as well as negative controls (no reverse transcription and no template controls). Data were analyzed with the analysis software provided with the Mic qPCR cycler that uses the REST tool for relative expression analysis (Pfaffl et al., 2002). Specifically, quantification cycle C_q_ values and reaction efficiencies were calculated using the LinRegPCR method and relative quantification of specific genes were determined using two reference genes, *rpoB* and *gyrB*. For RNA stability assays, the 16S rRNA gene was used as the reference gene. Each experiment was independently performed twice.

### Creation of a *lacZ* reporter system

To create a translational reporter system, the *lacZ* gene beginning at codon 9 was amplified (primers lacZ_9F+lacZ_R) from genomic DNA of *E. coli* strain MG1655 and inserted into *Hind*III-*Pst*I digested pME6031 (Heeb et al., 2000), creating plasmid pKM002. The *Kpn*I site in the MCS region was then inactivated by digestion with *Kpn*I and treatment with Klenow DNA polymerase followed by ligation, generating plasmid pKM202. The artificial promoter containing a Lac operator between the −35 and −10 motifs (created by annealing the two oligonucleotides PlacO_f and PlacO_r) was inserted into *Hind*III digested plasmid pKM202 thereby forming pKM232. The 5′ untranslated region (5′UTR) and the beginning of selected genes were amplified by PCR and cloned as *Kpn*I-*Hind*III fragments to construct translational fusions with *lacZ*. The transcription start site used was based on the data by Shao et al. (2014) and was located immediately after the *Kpn*I site in all constructs.

### β-galactosidase assay

Bacteria (Δ*ryhB* or Δ*hfq*) containing pKM232-derivatives and pKM033 or pKM033-ryhB were grown in LB medium supplemented with kanamycin and tetracycline. Over-night cultures were diluted to OD_600nm_ ~0.025 and incubated for 90 min at which time expression was induced by addition of 0.5 mM IPTG and cultures were grown an additional 90 min. One hundred microliters of bacterial culture was lyzed in 1 ml Z-bufffer (0.1M KCl, 1 mM MgSO_4_, 60 mM Na_2_HPO_4_, 40 mM NaH_2_PO_4_; pH 7) with 0.27% β-mercaptoethanol, 50 μl chloroform and 25 μl 0.1% SDS. After equilibration at 30°C, 200 μl o-nitrophenyl-β-D-galactopyranoside (ONPG, 4 mg/ml) was added and reactions were stopped when a yellow color developed by adding 500 μl 1M Na_2_CO_3_. Each strain was grown in duplicate with triplicate samples taken from each and the experiment was independently repeated twice. Specific activities were calculated as Miller units [1,000^*^(OD_420_)/(time^*^volume^*^OD_600_)] and shown as the mean ± *SD* of all samples.

## Results

### Fur and RyhB control growth and expression of heme-containing proteins

**Figure 1:**
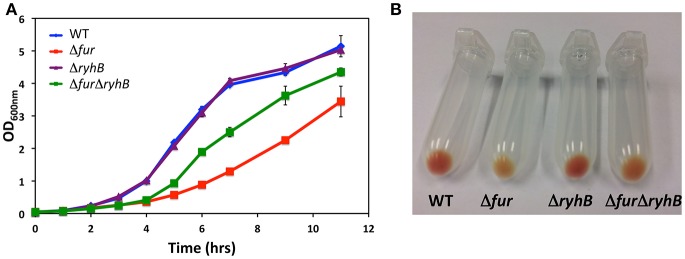
Deletion of *ryhB* nearly abolishes the phenotypes of a *fur* mutant. **(A)** Growth of *S. oneidensis* wild-type, Δ*fur*, Δ*ryhB*, and Δ*fur*Δ*ryhB* strains in LB medium at 30°C with shaking. **(B)** Bacterial pellets of stationary phase cultures of *S. oneidensis* wild-type and mutants.

A transposon screen in *S. oneidensis* identified a mutant with an insertion in the gene locus encoding the ferric uptake regulator Fur, resulting in decreased expression of the outer membrane multiheme *c*-type cytochrome MtrC (K. L. Meibom and R. Bernier-Latmani, unpublished data). Deletion of the *fur* gene in wild-type *S. oneidensis* showed that loss of Fur affected growth of *S. oneidensis* in liquid culture and the mutant formed colonies of smaller size than the wild-type strain (Figure 1A and Supplementary Figure 1A), in accordance with an earlier report (Yang et al., 2008). Complementation with a plasmid encoding *fur* restored colony size (Supplementary Figure 1B). It is well-known from studies in other bacterial species that even though Fur is generally a repressor, it can control expression of target genes positively through the sRNA RyhB (Oglesby-Sherrouse and Murphy, 2013). *S. oneidensis* MR-1 encodes a RyhB homolog between genes SO_4716 and SO_4717 and the gene appear to encode a ~127 nt transcript based on the previously identified transcription start site (Shao et al., 2014) and prediction of a rho-independent terminator using the tool ARNold (Naville et al., 2011; Supplementary Figures 2A,B). To study the role of RyhB in *S. oneidensis*, we deleted the gene in both the wild-type strain and in the Δ*fur* mutant strain. Northern blot analysis confirmed the expression of RyhB and the approximate size of the RNA (Supplementary Figure 2C). Deleting *ryhB* had no obvious phenotype in the wild-type strain, whereas deletion of *ryhB* in the Δ*fur* strain almost reversed the observed growth defect of the Δ*fur* strain (Figure 1A and Supplementary Figure 1A). Also, overproduction of RyhB from a plasmid had a negative effect on growth of the wild-type and Δ*ryhB* strains (Supplementary Figure 1C), indicating that the increased expression of RyhB in the Δ*fur* strain is responsible for this phenomenon. Another phenotype is the pale color of the colonies formed by the Δ*fur* strain compared to the reddish color of the wild-type *S. oneidensis* colonies. The reddish color is attributed to the high level of cytochromes in *S. oneidensis*. We observed the same phenotype in our mutant strain in liquid culture but it was more readily noticeable in bacterial pellets (Figure 1B). Again, the deletion of *ryhB* in the Δ*fur* mutant nearly reverted the phenotype to wild-type appearance (Figure 1B). Furthermore, heme-staining of cell lysates after polyacrylamide gel electrophoresis showed that the Δ*fur* mutant contains a lower level of heme-containing proteins and this can be nearly completely reversed by deleting *ryhB* (Supplementary Figure 1D). Taken together, these results suggest that the Fur protein, through the sRNA RyhB, controls expression of proteins with importance for growth and controls the production of heme-containing proteins.

### Identification of potential RyhB repressed genes

In order to identify targets of RyhB regulation in *S. oneidensis*, we used RNA sequencing (RNAseq) to assess the transcriptomes in various strains. We grew the wild-type, Δ*fur*, and Δ*fur*Δ*ryhB* strains in LB medium and isolated total RNA from three independent cultures of each. We hypothesized that Fur acts indirectly as a positive regulator via RyhB. According to the presumed regulatory mechanism, genes negatively regulated by RyhB should exhibit decreased expression in the Δ*fur* mutant, where RyhB levels are high relative to the wild-type strain. Conversely, we would expect an increased expression of a target gene in the Δ*fur*Δ*ryhB* mutant compared to the Δ*fur* mutant. The RNA sequencing confirmed that Fur acts as a repressor of *ryhB* expression as the RyhB level increases in the Δ*fur* mutant, as well as a repressor of the expression level of a number of other genes (Supplementary Table 3). Analysis of the RNAseq data showed that 64 genes were expressed at a lower level in the Δ*fur* mutant relative to the wild-type strain (minimum 2-fold difference and *p* < 0.05; Table 1).

Of the 64 genes we identified as expressed at a lower level in the Δ*fur* mutant relative to wild-type, 46 genes were expressed at a higher level in the Δ*fur*Δ*ryhB* mutant relative to the Δ*fur* mutant (*p* < 0.05), but were not differently expressed in the Δ*fur*Δ*ryhB* mutant relative to the wild-type strain (Table 1, group I). This result indicates that RyhB regulates these 46 genes and is responsible for the entire decrease in expression seen in the Δ*fur* mutant relative to the wild-type. A second subset of the 64 Fur-activated genes, consisting of 12 genes, was expressed at a significantly different level (*p* < 0.05) in the Δ*fur*Δ*ryhB* mutant relative to both the Δ*fur* mutant and to the wild-type strain (Table 1, group II). Specifically, these genes were expressed at a higher level in the Δ*fur*Δ*ryhB* mutant compared to the Δ*fur* mutant and at a lower level relative to the wild-type. This expression pattern suggests that RyhB regulates these genes, but that other regulators could contribute as well. Finally, 6 genes did not differ in expression level in the Δ*fur*Δ*ryhB* mutant compared to the Δ*fur* mutant (Table 1, group III and other), suggesting that Fur, but not RyhB regulates these genes.

To obtain a more direct measure of the targets of RyhB regulation, we performed RNAseq of total RNA isolated from a Δ*ryhB* mutant containing either a plasmid with an inducible *ryhB* gene or an empty plasmid. This allowed us to divide the 64 genes expressed at lower levels in the Δ*fur* strain into further subgroups. Of the 46 genes in group I, we found that 22 were expressed at a lower level when RyhB was overexpressed (Table 1, group Ia), strongly indicating that these genes are directly controlled by RyhB. Conversely, 24 genes belonging to group I did not change expression after overexpression of RyhB (group Ib). Of the group II genes, 8 out of 12 genes exhibited decreased expression when RyhB was overexpressed (group IIa), pointing to a direct control of these by RyhB.

Thus, genes from both group Ia and group IIa show a direct response to RyhB overexpression, indicating that they are regulated by Fur via RyhB. But, group Ia likely is regulated by Fur through RyhB exclusively, while group IIa is regulated by Fur through RyhB but also likely through another Fur-dependent or iron-dependent regulator.

We observe that groups Ia and IIa are comprised of many genes encoding iron-containing proteins such as cytochromes and proteins with iron-sulfur clusters. In fact, genes encoding iron-containing proteins represent about 50% of the RyhB-regulated genes (Table 1). Our data show that RyhB influences the expression of 10 of the 41 possible functional *c*-type cytochromes: *scyA, cctA, SO_3420, nrfA, cymA, petC, mtrA, mtrC, SO_4047*, and *SO_4666*. Membrane proteins and transporters, including Omp35 (SO_3896), account for most of the remaining RyhB regulated genes, in both group Ia and IIa.

It is also noteworthy, that one of the first genes known to be regulated by RyhB in *E. coli, sodB* encoding superoxide dismutase (Massé and Gottesman, 2002), is also regulated by this sRNA in *S. oneidensis* as was suggested by an earlier study (Yang et al., 2010).

One of the genes assigned to group III (genes not controlled by RyhB), SO_1776 encoding the outer membrane component MtrB of the MtrABC system for metal respiration, was actually downregulated by overexpression of RyhB (1.7-fold, *p* = 0.004). Indeed, revisiting the data showed that *mtrB* was expressed at a very slightly increased level in the Δ*fur*Δ*ryhB* mutant compared to the Δ*fur*, but not significantly (*p* = 0.059). In light of the RyhB overexpression data and that fact the *mtrB* is located within an operon with *mtrC* and *mtrA*, that both belong to group IIa, we believe *mtrB* is likely to belong to that group of genes as well.

Surprisingly, three group III genes that did not appear to be controlled by RyhB (based on the fact that the expression level did not increase when *ryhB* was deleted in the Δ*fur* mutant) had elevated expression when RyhB was overexpressed. The genes encode an inner membrane protein of unknown function (SO_1977) and bacterioferritin subunits 1 and 2, an iron-storage complex.

### Iron-availability controls expression of periplasmic *c*-type cytochromes ScyA and CctA

**Figure 2:**
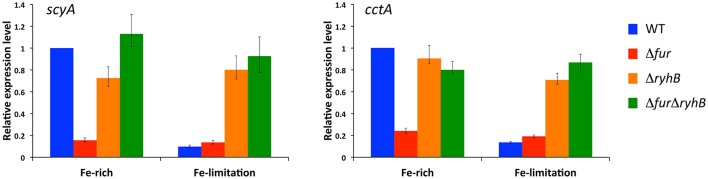
Expression of *scyA* and *cctA* is regulated by iron-availability through Fur and RyhB. Transcript levels were assessed by qRT-PCR in *S. oneidensis* wild-type, Δ*fur*, Δ*ryhB*, and Δ*fur*Δ*ryhB* strains in rich medium (iron-rich) and after 30 min growth with the iron chelator 2,2′-dipyridyl (iron-limitation). Transcript levels are shown relative to wild-type level in rich medium as expression ratio of four technical replicates (error bars, standard error). Results from an independent experiment are shown in Supplementary Figure 3.

Our RNAseq data suggested that two major periplasmic *c*-type cytochromes ScyA and CctA are indirectly activated by Fur, through RyhB. Because Fur responds to iron availability, we next assessed whether iron levels influence expression of *scyA* and *cctA* in the genetic backgrounds we constructed. Figure 2 (Supplementary Figure 3) show the relative expression values of *scyA* and *cctA* in the wild-type, Δ*fur*, Δ*ryhB*, and Δ*fur*Δ*ryhB* strains during growth in LB medium (Fe-rich) and 30 min after addition of the iron chelator 2,2′-dipyridyl (Fe-limitation). Iron-limitation dramatically decreased expression of both *scyA* and *cctA* in the wild-type strain, to about 10–20% of the level under iron-rich conditions. In contrast, iron depletion did only very slightly influence expression in the Δ*fur* mutant, where expression was at a low level under both conditions. This suggests that sensing of iron availability through Fur controls expression of these two cytochromes. Deletion of *ryhB* in the wild-type background had no or a very minor effect on *scyA* and *cctA* expression. Their expression level remained relatively high under iron limitation, demonstrating that RyhB is required for the decreased expression under iron limiting conditions. Likewise, expression levels of *scyA* and *cctA* in the Δ*fur*Δ*ryhB* mutant were not or only very slightly affected by iron-limitation and stayed at a level comparable to the wild-type strain under iron-rich conditions. Taken together, these data strongly indicate that iron limitation decreases expression of the periplasmic cytochromes ScyA and CctA in a Fur-dependent manner that relies on the presence of RyhB.

### RyhB alters the stability of several target transcripts

**Figure 3:**
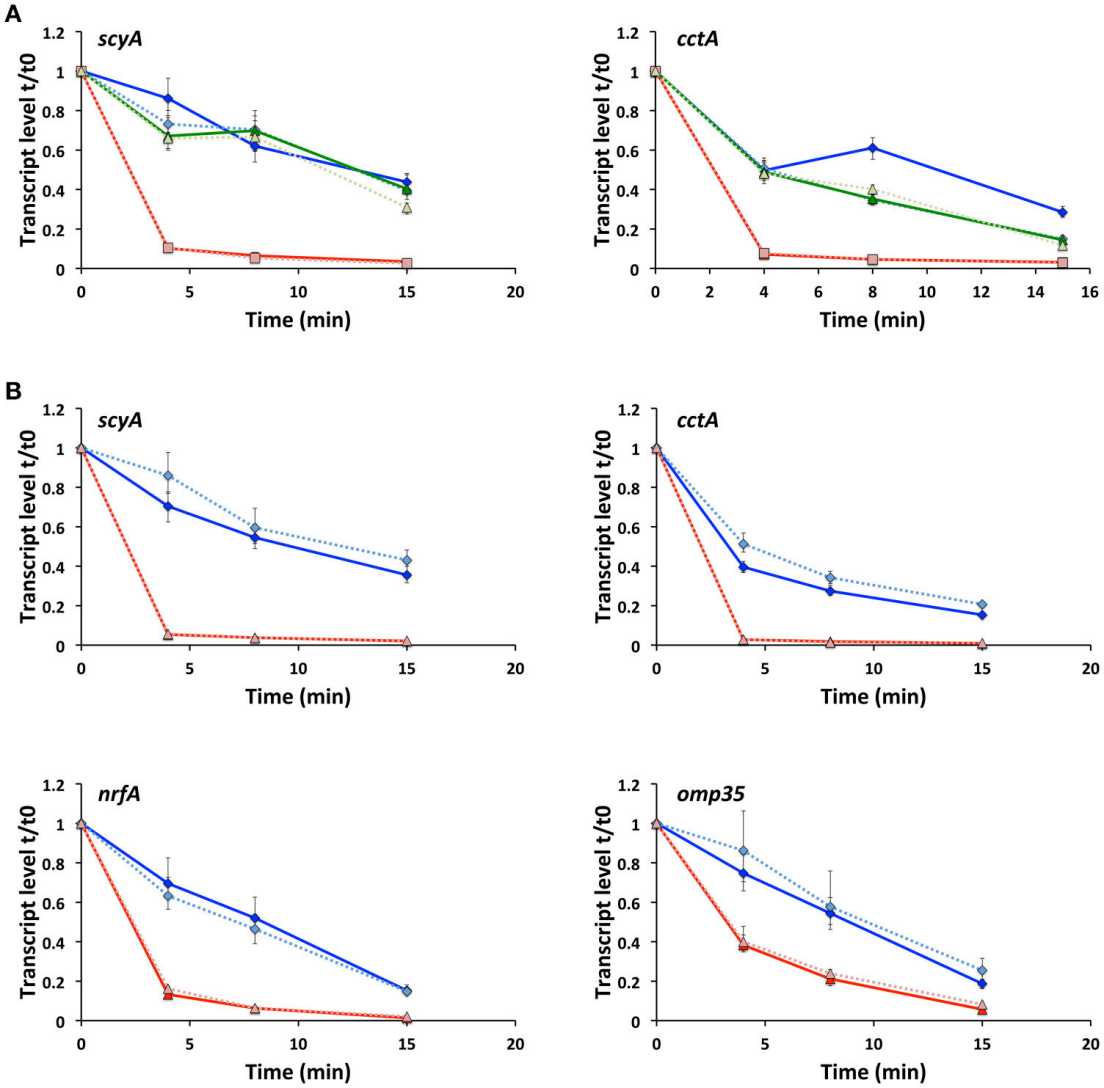
Production of RyhB affects the stability of the target mRNA. **(A)** Transcript levels of *scyA* and *cctA* genes in the wild-type (blue diamonds), Δ*fur* (red squares) and Δ*fur*Δ*ryhB* (green triangles) strains at 0, 4, 8, and 15 min after transcription was stopped. qRT-PCR was used to determine the transcript level relative to time 0, just before addition of rifampicin. Data shown are from two experiments; each with four replicate cDNA samples used in qPCR and shown as expression ratio ± standard error. For all strains, data from experiment 2 are depicted in lighter color and using a dashed line. **(B)** Transcript levels of *scyA, cctA, nrfA*, and *omp35* genes at 0, 4, 8, and 15 min after transcription was stopped in the Δ*ryhB* mutant containing an empty plasmid (pKM033) (blue diamonds) or a plasmid expressing RyhB (pKM033-ryhB) (red triangles). qRT-PCR was used to determine the transcript level relative to time 0, just before addition of rifampicin. Data are from two experiments; each with four replicate cDNA samples used in qPCR and shown as expression ratio ± standard error. For both strains, data from experiment 2 are depicted in lighter color and using a dashed line.

To establish whether RyhB regulates the expression of gene targets in *S. oneidensis* by affecting the stability of their mRNAs, we determined the transcript levels of *scyA* and *cctA* at specific times after transcription was stopped in the wild-type, Δ*fur* and Δ*fur*Δ*ryhB* strains (Figure 3A). We observed that the amount of transcript decreases in all strains over time, but dramatically faster in Δ*fur* than in the wild-type or Δ*fur*Δ*ryhB* strains, showing that RyhB affects transcript stability. When RyhB is overexpressed from a plasmid, it has a similar severe effect on transcript stability (Figure 3B), suggesting that the decreased transcript levels observed in the Δ*fur* strain, is due to mRNA destabilization rather than decreased transcription. We wondered whether this effect would also be observed with other of the RyhB regulated target genes we identified by RNAseq analysis. As can be seen in Figure 3B, both *omp35* and *nrfA* transcript stabilities were significantly altered after RyhB overexpression. In contrast, this was not the case for the RyhB-independent *recA* gene, verifying that overexpression of RyhB does not generally affect mRNA stability in *S. oneidensis* (Supplementary Figure 4). The results show that RyhB affect the mRNA stability of its targets ScyA, CctA, NrfA, and Omp35 and suggest that RyhB binding to the target mRNAs promotes degradation by RNases.

### RyhB down-regulation of cytochromes CctA and ScyA is mediated by the 5′-end of target genes

**Figure 4:**
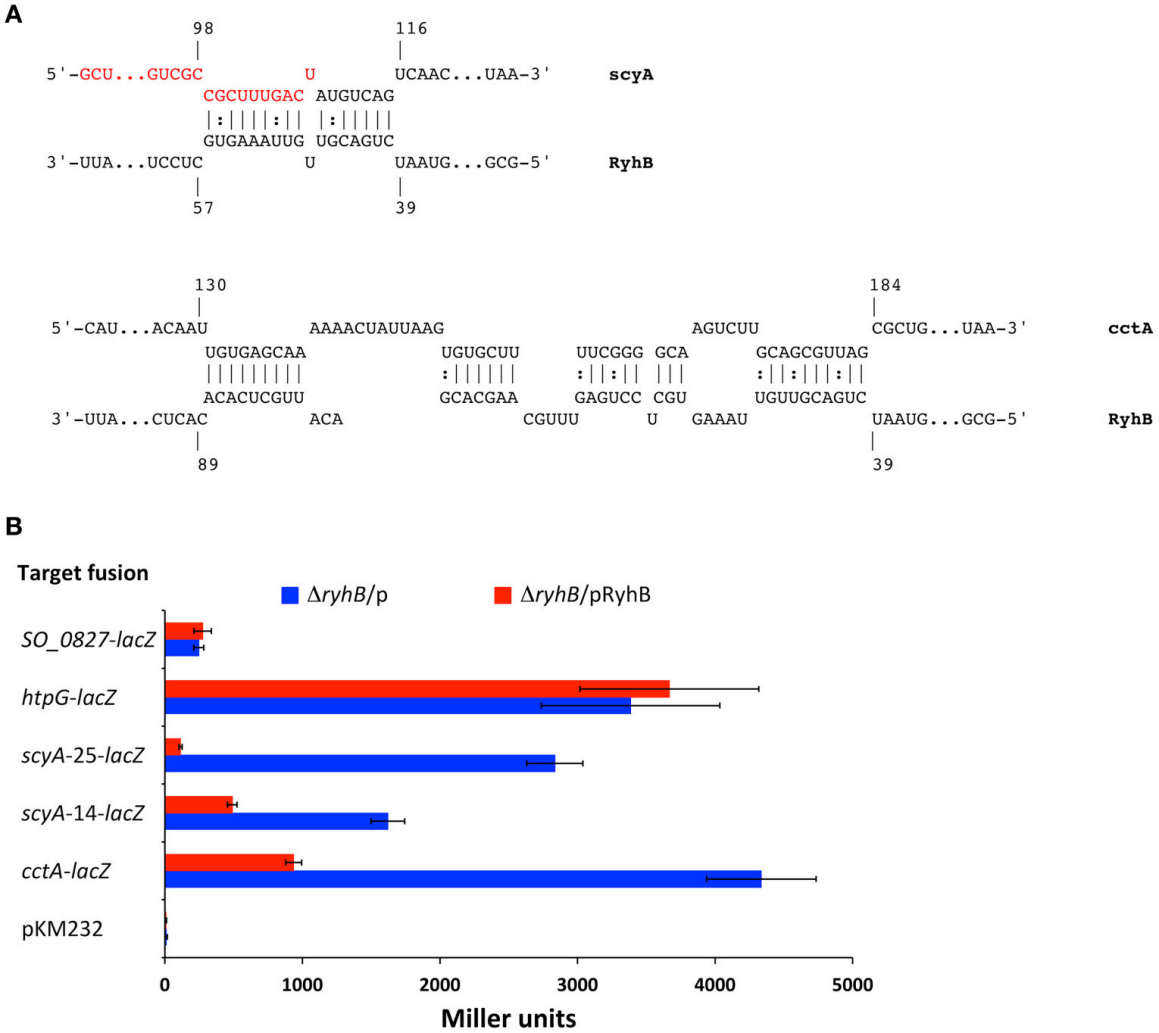
RyhB regulation of ScyA and CctA expression is mediated by sequences in the start of the genes. **(A)** Predicted interactions between RyhB and *scyA* and *cctA* mRNAs. Predictions were made with IntaRNA (Busch et al., 2008; Wright et al., 2014; Mann et al., 2017). Nucleotides are numbered according to their transcription start site (Shao et al., 2014). The transcription start site is located 66 nt upstream from the translational start for *scyA. cctA* contains two transcription start sites located 85 and 131 nt upstream from the translational start site, respectively (the numbers in the figure refer to the start site 131 nt upstream the translational start, which is used for the *cctA-lacZ* fusion). Nucleotides marked in red are designating the end of *scyA* codons in the fusion *scyA*-14*-lacZ*. **(B)** Regulation of target gene fusions by expression of RyhB. β-galatosidase activity was quantified from gene-*lacZ* fusions harbored on a plasmid in the presence of another plasmid with inducible RyhB production, pKM033-ryhB (pRyhB), or in the presence of an empty plasmid, pKM033 (p). pKM232 contains a promoter-less and truncated *lacZ* gene missing the first 8 codons. Data are from two independent experiments, each with duplicate cultures, and triplicate samples. Activity is expressed as the mean (Miller units) ± *SD*.

Our data show that RyhB down-regulates expression of the *cctA* and *scyA* genes by affecting their mRNA stability. *In silico* analysis using the IntaRNA software (Busch et al., 2008; Wright et al., 2014; Mann et al., 2017) predicts that RyhB interacts with *scyA* mRNA sequences at the beginning of the coding region but down-stream from the ribosome binding site and start codon (Figure 4A). RyhB is predicted to interact with a relatively long sequence of the *cctA* mRNA beginning at the start codon. In order to investigate whether RyhB interacts with the 5′end of these two target genes and to present further evidence for RyhB-dependent regulation of these genes, we established a *lacZ*-based reporter system in *S. oneidensis*. The 5′UTR (transcription start sites are designated as +1 and are based on Shao et al., 2014) of *cctA* and *scyA* and the beginning of the genes were fused in-frame to the *E. coli lacZ* missing the first 8 codons. The fusions were expressed on a plasmid from an artificial P_lac_ promoter containing a LacI binding site between the −10 and −35 sequences. At the same time, RyhB is expressed from the same promoter on a second plasmid, which also contains the *lacI* gene from *E. coli*. The *cctA-lacZ* fusion comprises the first 26 codons of *cctA*, which contains the entire sequence predicted to interact with RyhB. We made two different fusions of *scyA* with *lacZ*, one containing the first 25 codons of *scyA*, which include the entire sequence predicted to interact with RyhB (*scyA*-25-*lacZ*) and a second encompassing only part of the interaction region (*scyA*-14-*lacZ*) (Figure 4A). When the fusions were expressed in the Δ*ryhB* strain in combination with the plasmid expressing RyhB, the activity of the fusion protein was lower than when RyhB was not expressed (Figure 4B). Even though the activity of both *scyA* fusions were lower in the presence of RyhB, the fusion containing the entire region predicted to interact with RyhB was decreased to a significantly higher extent (25- vs. 3.3-fold decrease). This indicates that the predicted region is involved in RyhB-dependent regulation and that the entire sequence is required for high-level regulation. No RyhB-dependent decrease in the activity of two control fusions was observed (*SO_0827-lacZ* and *htpG-lacZ*) ruling out a general effect of overproducing RyhB on LacZ activity. Thus, the reporter fusion data show that the 5′ ends of the *cctA* and *scyA* mRNAs are sufficient to ensure down-regulation of expression by RyhB and provide further evidence of RyhB-dependent down-regulation of the two cytochromes.

### Hfq is necessary for RyhB-dependent regulation

**Figure 5:**
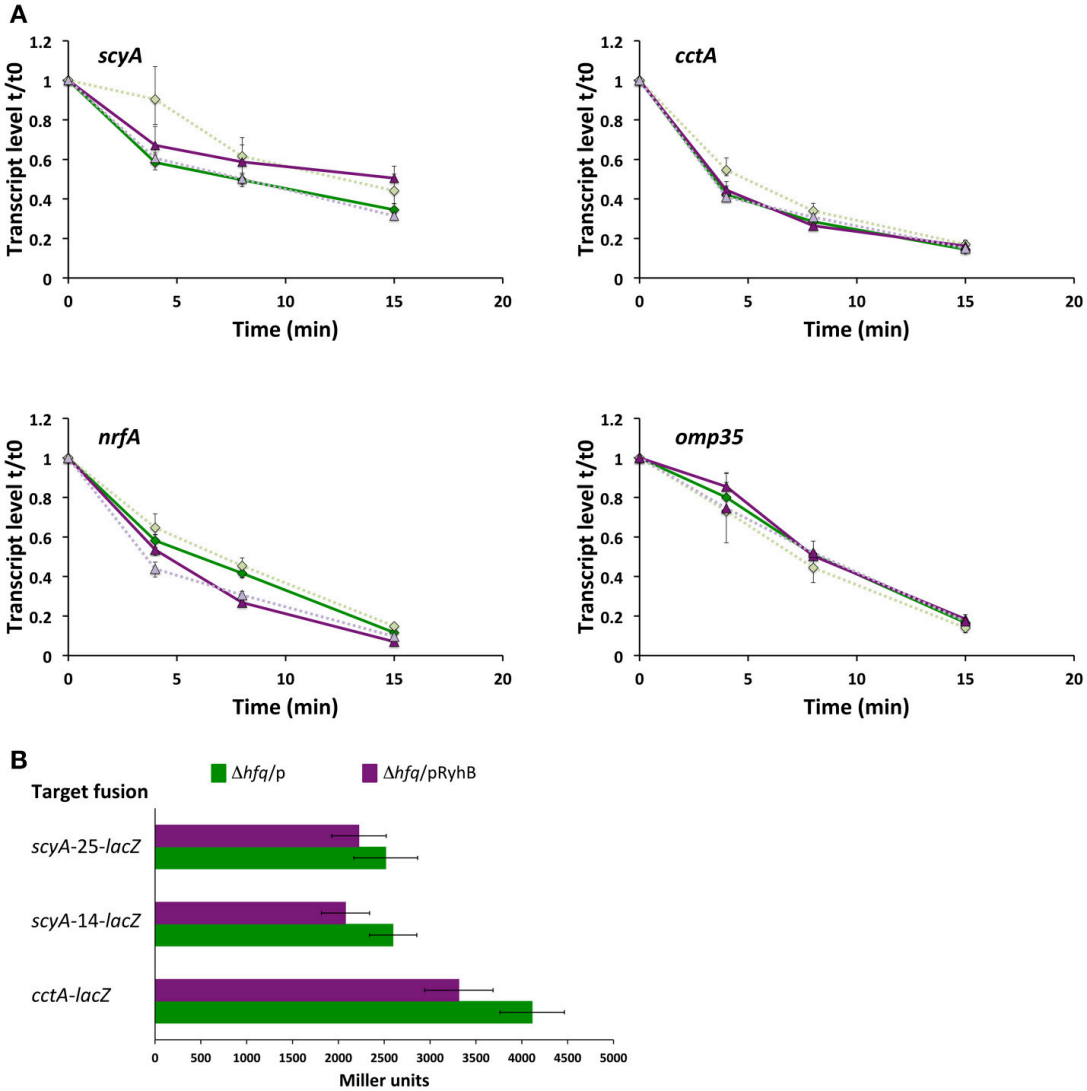
Hfq is required for regulation of RyhB targets. **(A)** Transcript levels of *scyA, cctA, nrfA*, and *omp35* genes at 0, 4, 8, and 15 min after transcription was stopped in a Δ*hfq* mutant containing an empty plasmid (pKM033) (green diamonds) or a plasmid expressing RyhB (pKM033-ryhB) (purple triangles). qRT-PCR was used to determine the transcript level relative to time 0, just before addition of rifampicin. Data are from two experiments; each with four replicate cDNA samples used in qPCR and shown as expression ratio ± standard error. For both strains, data from experiment 2 are depicted in lighter color and using a dashed line. **(B)** Activity of plasmid encoded target gene fusions in the Δ*hfq* mutant expressing RyhB (from pKM033-RyhB; pRyhB) or containing an empty plasmid (pKM033; p). Activities are shown as the mean (Miller units) ± *SD* and are the data from two independent experiments, each with duplicate cultures, and triplicate samples.

In Gram-negative bacteria, regulation of gene expression by sRNAs often relies on the RNA chaperone Hfq. To determine whether Hfq is required for RyhB function, we constructed a Δ*hfq* mutant strain, which exhibited the small colony and reduced growth phenotype that is well-known for several bacterial species, including *S. oneidensis* (Dietrich et al., 2009; Meibom et al., 2009; Mégroz et al., 2016). To assess the importance of Hfq for RyhB-dependent destabilization of target mRNAs, we overproduced RyhB in the Δ*hfq* mutant and quantified the mRNA level over time after transcription was stopped. Overproduction of RyhB either did not affect or very slightly affected the stability of the mRNA of the target genes *scyA, cctA, nrfA*, and *omp35* (Figure 5A), which is in stark contrast to the results obtained when Hfq is present (Figure 3B). This finding points to the fact that Hfq is required for the RyhB-induced destabilization of the target mRNAs. The involvement of Hfq was further assessed by measuring the activity of the gene-*lacZ* fusions in the Δ*hfq* strain (Figure 5B). The activity of the *cctA-lacZ* and both *scyA-lacZ* fusions decreased only slightly when RyhB was overproduced in this mutant. From this evidence, we conclude that Hfq is required for optimal function of RyhB during regulation of CctA and ScyA expression.

## Discussion

*S. oneidensis* uses numerous electron acceptors for respiration. Most electron acceptors are reduced in the periplasm or at the bacterial surface, with *c*-type cytochromes playing a key role. It has been suggested that the periplasmic electron transfer network is dynamic and that simultaneous production of several functional electron transport chains allows *S. oneidensis* to release electrons quickly to a variety of available electron acceptors (Sturm et al., 2015). The findings presented here strongly indicate that the expression of numerous *c*-type cytochromes with varying cellular locations and functional roles in response to fluctuating iron availability is regulated by the small RNA RyhB through the master iron regulator Fur.

The presence of a RyhB homolog in *S. oneidensis* was reported in a previous study that was primarily directed at determining RyhB's potential role in regulating TCA cycle genes (Yang et al., 2010). However, due to unsuccessful attempts to generate a null mutant, a specific role for RyhB was not demonstrated, although it was shown that RyhB expression was increased in a *fur* mutant and that RyhB overexpression reduced the amount of the *sodB* transcript, a known RyhB target in *E. coli*. Our results corroborate the findings that *sodB*, but not the TCA cycle genes *sdhA* and *acnA*, are targets of RyhB (Yang et al., 2010). Also, since we were successful in generating a *ryhB* mutant in both the wild-type strain and in a Δ*fur* mutant, we were able to characterize this sRNA in *S. oneidensis*.

RNA sequencing of mutant strains and a RyhB overexpressing strain permitted us to identify putative targets of RyhB regulation. The RNAseq results of the Δ*fur* mutant identified 64 genes downregulated in the mutant relative to wild-type (Table 1). It should be noted that other microarray-based early transcriptomic studies of a Δ*fur* mutant yielded disparate results (Thompson et al., 2002; Wan et al., 2004). The most recent study (Yang et al., 2008) has focused on genes upregulated in the Δ*fur* mutant relative to wild-type. We observed very little overlap between the 64 genes expressed at a lower level in our study, and the 56 expressed a lower level in Δ*fur* in the aforementioned study. This discrepancy is likely due to differing experimental conditions.

Amongst the 64 genes identified above, a sizeable number whose expression level is reduced by the action of RyhB encode proteins that contain iron as a cofactor. In *E. coli*, RyhB ensures iron conservation in order to guarantee its availability for essential pathways by reducing the expression of iron-binding proteins under iron-limiting conditions (Massé et al., 2005). A recent study found that heme biosynthesis enzymes, cytochrome maturation functions, and heme proteins are one group of iron-containing proteins that appear to mostly escape regulation by RyhB in *E. coli* (Beauchene et al., 2015). In contrast to *E. coli*, heme proteins make up the major part of the iron-containing proteins regulated by RyhB in *S. oneidensis* MR-1. Of the 41 possible functional *c*-type cytochromes in *S. oneidensis*, RyhB negatively affected expression of 10: ScyA, PetC, MtrA, MtrC, CctA, SO_3420, NrfA, SO_4047, CymA, and SO_4666. We found that expression of these 10 cytochromes was reduced in a *fur* mutant (where RyhB levels are elevated) and when RyhB is over-expressed, and conversely that transcript levels increased when *ryhB* was deleted in the *fur* mutant. Since RyhB expression is repressed by Fur, its level increases under iron-limiting conditions when less Fur is actively bound to promoter regions. Therefore, expression of RyhB repressed genes is expected to decrease when iron becomes scarce. We confirmed this for *scyA* and *cctA* and would assume that other RyhB regulated cytochrome genes respond the same way. In fact, an earlier study of the iron response of *S. oneidensis* MR-1 found that iron depletion represses expression of a number of genes involved in anaerobic energy metabolism, including *cymA, mtrA, mtrC*, and *omcA* (Yang et al., 2009). Our results demonstrate that the previously observed down-regulation of cytochrome gene expression after iron depletion, with the exception of *omcA*, is likely to be mediated through Fur and RyhB.

A significant number of the genes in groups I and II were not downregulated after RyhB overexpression (groups Ib and IIb). This suggests that RyhB is not directly regulating these genes. In contrast, deleting *ryhB* in the Δ*fur* strain increased their expression, suggesting a role for RyhB. We have no conclusive explanation for these apparently contradictory results, but believe it could be a consequence of the effect deleting *fur* has on the physiology of *S. oneidensis*. Down-regulation of certain genes in the Δ*fur* mutant relative to the wild-type could be a result of the growth change tied to Fur loss, whereas the increase in expression after the consecutive deletion of *ryhB* reflects the reversion of growth to wild-type level rather than direct regulation by RyhB.

Three genes that, based on RNAseq data, were not regulated by RyhB (i.e., pertaining to group III) exhibited higher expression upon RyhB overexpression. This result can be attributed to increased intracellular iron concentrations as a result of the decreased production of iron-containing proteins, as observed in *E. coli* (Jacques et al., 2006). Increased iron concentration would lead to higher activity of Fur as a positive regulator of these genes. We do not know whether Fur directly activates these genes or whether another regulatory molecule, maybe another small RNA, is involved. In agreement with this, we found that some of the Fur-repressed genes (e.g., iron uptake genes that are increased in expression in the Δ*fur* strain) are transcribed at a lower level when RyhB is over-expressed (data not shown) and attribute this to elevated iron levels leading to increased repression by Fur.

We saw, as others before (Yang et al., 2008), that deletion of *fur* results in several physiological changes, most notably a decreased growth rate and loss of the signature red color of *S. oneidensis* cultures and colonies. Notably, these phenotypes were almost completely reversed by deletion of *ryhB*, emphasizing that RyhB controls important cellular processes. The disappearance of the red color can be explained by the significant downregulation of the numerous *c*-type cytochromes in the *fur* mutant, which was further supported by visualizing the reduced amount of heme-containing proteins in the Δ*fur* strain. It is also possible that the reduced growth of the Δ*fur* mutant is linked to the decreased production of cytochromes. Two of the most abundant periplasmic *c*-type cytochromes, ScyA and CctA, as well as the membrane anchored CymA, which functions as the electron donor to several periplasmic electron chains are significantly downregulated in the Δ*fur* mutant. It is known that deletion of *cymA* results in decreased ability to use the anaerobic electron acceptors Fe(III), Mn (IV), Cr(VI), nitrate, fumarate, and DMSO, but that it does not impact aerobic growth (Myers and Myers, 1997; Schwalb et al., 2003; Gao et al., 2010). The precise role played by ScyA is not known, but it has been identified as a mediator of electron transport between CymA and the diheme cytochrome c5 peroxidase CcpA (Schütz et al., 2011). Whereas a *scyA* null mutant does not exhibit any major phenotype with respect to growth with a number of electron acceptors (Jin et al., 2013), the gene does appear to have an impact on the fitness of *S. oneidensis* under some conditions (Deutschbauer et al., 2011; Wetmore et al., 2015).

The genes for the three subunits of the ubiquinol-cytochrome *c* reductase complex are all expressed at lower level in the *fur* mutant, and deleting *ryhB* abolishes this. The three genes encode the Fe-S subunit PetA, the cytochrome *b* subunit PetB, and the cytochrome *c*1 subunit PetC. It is possible that the decreased expression of these genes accounts for the growth defect of the *fur* mutant as both *petA* and *petC* mutants exhibit an aerobic growth defect (Gao et al., 2010; Luo et al., 2013).

This study was initiated by searching for regulators controlling expression of the outer membrane decaheme *c*-type cytochrome MtrC, one of the proteins involved in metal respiration. Our RNA sequencing data strongly indicate that MtrC is controlled by Fur, indirectly by the action of the sRNA RyhB. However, the data also suggest that other regulators contribute, as *ryhB* deletion does not completely relieve the effect of the *fur* deletion. Under iron-limiting conditions, when expression of RyhB is increased, *mtrC* transcript levels decrease, however the *mtrC* transcript level is also decreased under iron-limitation in a *ryhB* mutant (data not shown), supporting that other iron-dependent regulators in addition to RyhB control *mtrC* expression. The identity of this regulator (or regulators) is currently unknown.

In other species, RyhB exerts its function by base-pairing to sequences in target gene mRNAs, which often leads to mRNA degradation (Oglesby-Sherrouse and Murphy, 2013). Hfq is a RNA chaperone that is required for the function of many regulatory sRNAs (Updegrove et al., 2016). Hfq mediates interactions between sRNA molecules and their mRNA targets and stabilizes the sRNA. Since Hfq participates to the function of numerous sRNAs in a single organism, *hfq* null mutants often exhibit pleiotropic phenotypes. This is also the case for *S. oneidensis* (Brennan et al., 2013) and specifically it was observed that a *hfq* mutant synthesizes hemes at a reduced level, although the mechanism remains unknown (Brennan et al., 2014). Our data demonstrate that RyhB requires Hfq for its function in *S. oneidensis*. Over-production of RyhB strongly destabilizes the mRNA of the examined targets, *cctA, scyA, nrfA*, and *omp35* but this is circumvented in the *hfq* null mutant. We assume Hfq promotes limited base-pairing between RyhB and the target mRNAs. This is supported by assessing the effect of RyhB over-production on the activity of fusions between *lacZ* and the mRNA region of *scyA* and *cctA* that was proposed *in silico* to base-pair with RyhB. When Hfq is present, RyhB over-production reduces the activity of the reporter fusions but no decrease was seen when Hfq is absent. As Hfq appears to be required for RyhB function, we would expect the effect on growth observed due to loss of *fur* that can be alleviated by deleting *ryhB*, could also be alleviated by deletion of *hfq*. However, despite numerous attempts, we were unable to construct a double *fur hfq* null mutant, possibly due the effect the loss of each gene has on the growth.

**Figure 6:**
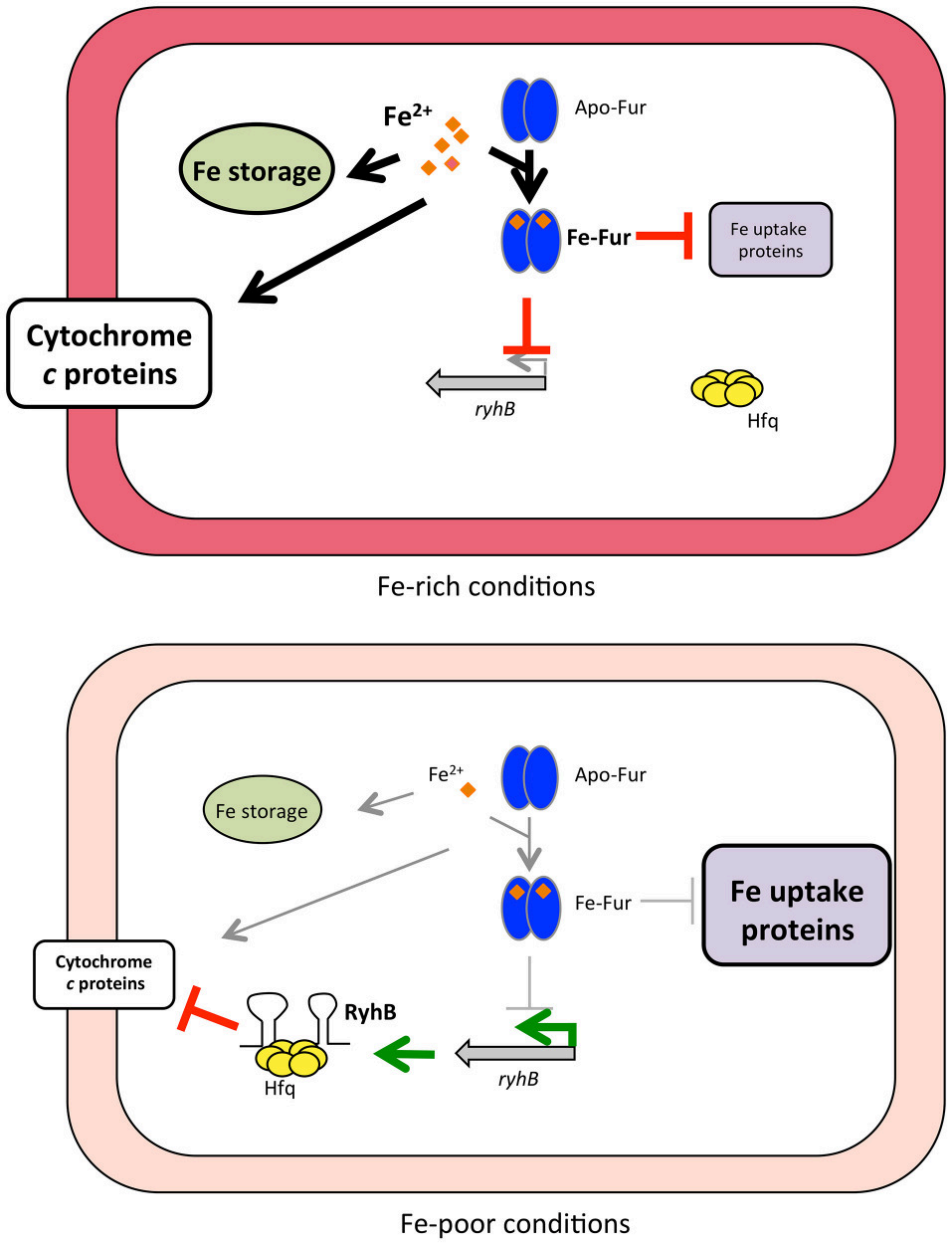
Model of how iron availability controls RyhB regulation of *c*-type cytochromes. Under iron-rich conditions, Fur associates with Fe^2+^ (orange diamonds) and binds to Fur-binding sites in the promoter region of target genes, leading to repression of expression (indicated by red blocked lines) of RyhB and other genes (e.g., iron uptake genes). Fur repression is relieved when iron becomes limiting and RyhB is produced (indicated by green arrows), as well as other Fur-repressed genes (including iron uptake genes). RyhB with the aid of the RNA chaperone Hfq represses expression of numerous *c*-type cytochromes by promoting degradation of the mRNAs. Processes that are favored under either Fe-rich or Fe-poor conditions are shown with thick arrows or thick blocked lines, whereas processes that do not occur (or at diminished rate) are indicated with thin gray arrows or thin gray blocked lines.

In summary, we propose a model for regulation of how iron availability controls cytochrome expression through Fur and the sRNA RyhB in *S. oneidensis* (Figure 6). Under iron-replete conditions, Fur represses of expression of RyhB as well as many proteins involved in iron uptake and *c*-type cytochromes are expressed. Iron limitation alleviates Fur repression, thus RyhB expression is increased and this reduces production of numerous cytochromes, thereby likely preserving iron for essential proteins. Our data furthermore suggest that some genes, including *mtrC*, rely not only on RyhB, but also on additional Fur- or iron-dependent molecules(s) for their regulation.

## Author contributions

KM designed the research, performed the experiments, analyzed, and interpreted the data, and wrote the manuscript. EC analyzed sequencing data and wrote part of the manuscript. RB-L designed the research interpreted results and wrote the manuscript.

### Conflict of interest statement

The authors declare that the research was conducted in the absence of any commercial or financial relationships that could be construed as a potential conflict of interest.
