# Supplementary material for: The Small RNA RyhB Is a Regulator of Cytochrome Expression in Shewanella oneidensis
Source: Front Microbiol. 2018 Feb 21;9:268. doi: 10.3389/fmicb.2018.00268 (PMC5826389; doi:10.3389/fmicb.2018.00268)
Supplement: Supplementary file 1 [file Table1.PDF]

Supplementary Material

The small RNA RyhB is a regulator of cytochrome expression in *Shewanella oneidensis*

Karin L. Meibom[1*], Elena M. Cabello[2], Rizlan Bernier-Latmani[1]

*** Correspondence: Karin L. Meibom**: karin.meibom@epfl.ch

Supplementary Table 1. Bacterial strains and plasmids

Strain or plasmid	Description	Source
Escherichia coli		
DH5α	F⁻ Φ80*lacZ*ΔM15 *endA1 recA1 hsdR17 supE44-thi-1 gyrA96 relA1* (*lacZYA*-argF)*U169*l	Lab collection
DH5αλ*pir*	F⁻ ϕ80*lacZ*ΔM15 *endA1 recA1 hsdR17 supE44-thi-1 gyrA96 relA1* (*lacZYA*-argF)*U169*l λ*pir* lysogen	A. Charbit
WM3064	*thrB*1004 *pro thi rpsL hsdS lacZ*ΔM15 RP-4-1360 Δ(*araBAD*)567 Δ*dapA*1341::[*erm pir*(wt)]	S.B. Reed
MG1655	K-12 wild-type strain	V. Sentchilo
Shewanella oneidensis		
MR-1	wild-type	Lab collection
MR-1Δf*ur*	MR-1 with markerless deletion of *fur*	This study
MR-1Δ*ryhB*	MR-1 with markerless deletion of *ryhB*	This study
MR-1Δ*fu*rΔ*ryhB*	MR-1 with markerless deletions of *fur* and *ryhB*	This study
MR-1Δ*hfq*	MR-1 with markerless deletion of *hfq*	This study
BG148	MR-1 with transposon insertion in *ccmC*	(Bouhenni et al., 2005)
Plasmids		
pMQ150	Yeast recombineering vector (*sacB oriT R6K nptII rpsL ura3 cen6*)	(Shanks et al., 2009)
pMQS	Mobilizable suicide vector, Km^r (*sacB oriT R6K nptII cen6*)	This study

pMQSΔfur	pMQS with regions flanking *fur*	This study
pMQSΔryhB	pMQS with regions flanking *ryhB*	This study
pMQSΔhfq	pMQS with regions flanking *hfq*	This study
pHGE-P*tac*	Broad-host expression vector with P_{tac} promoter, Km^r	(Luo et al., 2013)
pHGE-P*tac-fur*	pHGE-P*tac* containing *S. oneidensis fur* gene	This study
pBAD-fccA-His	Expression vector with *S. oneidensis fccA* gene under control of P_{BAD} promoter, Km^r	(Schuetz et al., 2009)
pET28b(+)	Expression vector, Km^r	Lab collection
pKM033	Vector with artificial P_{Laco-1} promoter and *E. coli lacI* gene, Km^r	This study
pKM033-ryhB	pKM033 containing *S. oneidensis ryhB* gene	This study
pME6031	Broad-host plasmid, Tc^r	(Heeb et al., 2000)
pKM002	pME6031 with promoter-less and N-terminal truncated *lacZ* gene	This study
pKM202	pKM002 with inactivated KpnI site	This study
pKM232	pKM202 with artificial P_{Laco-1} promoter	This study
pKM232-5'UTR-htpG	pKM232 with 5'UTR and first 15 codons of *htpG*	This study
pKM232-5'UTR-SO_0827	pKM232 with 5'UTR and first 24 codons of *SO_0827*	This study
pKM232-5'UTR-cctA	pKM232 with 5'UTR and first 26 codons of *cctA*	This study
pKM232-5'UTR-scyA-14	pKM232 with 5'UTR and first 14 codons of *scyA*	This study
pKM232-5'UTR-scyA-25	pKM232 with 5'UTR and first 25 codons of *scyA*	This study

Bouhenni, R., Gehrke, A., and Saffarini, D. (2005). Identification of genes involved in cytochrome *c* biogenesis in *Shewanella oneidensis*, using a modified mariner transposon. Appl. Environ. Microbiol. *71*, 4935–4937.

Heeb, S., Itoh, Y., Nishijyo, T., Schnider, U., Keel, C., Wade, J., Walsh, U., O'Gara, F., and Haas, D. (2000). Small, stable shuttle vectors based on the minimal pVS1 replicon for use in gram-negative, plant-associated bacteria. Mol. Plant-Microbe Interact. MPMI *13*, 232–237.

Luo, Q., Dong, Y., Chen, H., and Gao, H. (2013). Mislocalization of Rieske protein PetA predominantly accounts for the aerobic growth defect of Tat mutants in *Shewanella oneidensis*. PloS One *8*, e62064.

Schuetz, B., Schicklberger, M., Kuermann, J., Spormann, A.M., and Gescher, J. (2009). Periplasmic electron transfer via the *c*-type cytochromes MtrA and FccA of *Shewanella oneidensis* MR-1. Appl. Environ. Microbiol. *75*, 7789–7796.

Shanks, R.M.Q., Kadouri, D.E., MacEachran, D.P., and O'Toole, G.A. (2009). New yeast recombineering tools for bacteria. Plasmid *62*, 88–97.
