# Supplementary material for: The Small RNA RyhB Is a Regulator of Cytochrome Expression in Shewanella oneidensis
Source: Front Microbiol. 2018 Feb 21;9:268. doi: 10.3389/fmicb.2018.00268 (PMC5826389; doi:10.3389/fmicb.2018.00268)
Supplement: Supplementary file 2 [file Table2.PDF]

Supplementary Material

The small RNA RyhB is a regulator of cytochrome expression in *Shewanella oneidensis*

Karin L. Meibom[1*], Elena M. Cabello[2], Rizlan Bernier-Latmani[1]

*** Correspondence: Karin L. Meibom**: karin.meibom@epfl.ch

Supplementary Table 2. Oligonucleotides used in this study

**Oligonucleotide**	**Sequence (5'-3')[a]**	**Use**
Dfur_5F	CGGGGTACCGCCGTTATTCACTCCAAAAGG	Deletion of *fur*
Dfur _5R	TCCATCTGTCATTGCTAATCTC	Deletion of *fur*
Dfur_3F	GAGATTAGCAAATGACAGATGGACACAACGACGAATAAGCTTGC	Deletion of *fur*
Dfur_3R	CGGAATTCGTGTAGAGGGCGGTAAGGA	Deletion of *fur*
DryhB_5F	CCGGAATTCCAAATTCCTGAACTCGACCG	Deletion of *ryhB*
DryhB_5R	CATCAGAGTTAAAGATGAGTTTTG	Deletion of *ryhB*
DryhB_3F	CAAAACTCATCTTTAACTCTGATGCAGCGAATAGGCGCATTAGG	Deletion of *ryhB*
DryhB_3R	CGCGGATCCGTATTAGTGCGACATTCGATTC	Deletion of *ryhB*
Dhfq_5F	GAAAGAATTCATCCTAATGATCC	Deletion of *hfq*
Dhfq_5R	CCCCTTAGCCATTTTATTATTTC	Deletion of *hfq*
Dhfq_3F	GAAATAATAAAATGGCTAAGGGGGGCGAATAAGGTTTAGTTAAGG	Deletion of *hfq*

Dhfq_3R	CGCGGATTCCCTGAGCAAACGCCTATCG	Deletion of *hfq*
fur_FO	CACAGTTATCGAGCGCATC	Verification of *fur* deletion
fur_RO	GGAACGATGGTAAAAGTCTG	Verification of *fur* deletion
ryhB_FO	AGCAATGACAGCTCTGACTC	Verification of *ryhB* deletion
ryhB_RO	GAGTTCAATGCTCCGTTAGAG	Verification of *ryhB* deletion
hfq_FO	GCACTGCACGATCAATTAAGG	Verification of *hfq* deletion
hfq_RO	GGCGACTCTGCTCACGTTG	Verification of *hfq* deletion
fur_start	CGGAATTCATGACAGATGGAAATCAAGCG	Cloning of *fur* into pHGE-P*tac*
fur_stop	CGGAATTCTTATTCGTCGTTGTGCTCGCAG	Cloning of *fur* into pHGE-P*tac*
ELacI_F	TCCCCCGGGGACACCATCGAATGGCGCA	Cloning of *lacI* for creating pKM033
ELacI_R	TCCCCCGGGGCCTAATGAGTGAGCT	Cloning of *lacI* for creating pKM033
PlacO_f	AGCTATTATAAATGTGAGCGGATAACATTGACATTGTGAGCGGATAACAAGATACTGGATCCGGTACCA	Artificial promoter
PlacO_r	AGCTTGGTACCGGATCCAGTATCTTGTTATCCGCTCACAATGTCAATGTTATCCGCTCACATTTATAAT	Artificial promoter

ryhB_F(+1)	CGCGGATCCGCGTTCCAAAACTCATCTTTAA	Cloning of *ryhB* into pKM033
ryhB_R_Stop	CCCCAAGCTTCTATTCGCTGAATAACAGGCAA	Cloning of *ryhB* into pKM033
lacZ_9F	GCCCAAGCTTGCTGCCGTCGTTTTACAACGTCG	Cloning of *lacZ* into pME6031
lacZ_R	AACTGCAGTTATTATTTTTGACACCAGACCA	Cloning of *lacZ* into pME6031
ryhB_NB	bio-GAGTGTGTGAGCAATGTCGTGCTTGCAAACTCAGGAGCAC	Northern blot
gyrB_F_RT	GGCGTTCCTGATGCGCCAAT	qRT-PCR
gyrB_R_RT	CACGCACGCGTTTAGCCAGA	qRT-PCR
rpoB_F_RT	CAGCGGCCGGCACTGTAAAA	qRT-PCR
rpoB_R_RT	AAGAATACGCCTGGGCTGCG	qRT-PCR
scyA_F_RT	GCAGTCGCCGCTTTGACTATGT	qRT-PCR
scyA_R_RT	ACGTGGTTCCCAGTCAGCAGT	qRT-PCR
cctA_F_RT	CGCTGTCGCCAACCGCATT	qRT-PCR
cctA_R_RT	GCACCGTCAGCAGAAGGGGTA	qRT-PCR

16S_F_RT	CCATGCCGCGTGTGTGAAGA	qRT-PCR
16S_R_RT	GCTGCTGGCACGGAGTTAGC	qRT-PCR
recA_F_RT	AACCCGTTCTGGTGCGGTTG	qRT-PCR
recA_R_RT	ACGAGCCGCTAAGCCCATGT	qRT-PCR
nrfA_F_RT	GGCTGTAGCGATTGCCACGAA	qRT-PCR
nrfA_R_RT	CAACGTGACACTGGGCGCAT	qRT-PCR
omp35_F_RT	ACGCAGGTACTGTGTTAGTGGGT	qRT-PCR
omp35_R_RT	TCTGCGCTACGGGTTTGTGC	qRT-PCR
scyA_F(+1)	CGGGGTACCGCTATAATGAGTCTGCCTCAA	*scyA*-*lacZ* fusions in pKM232
scyA_R14	CCCCAAGCTTAGTCAAAGCGGCGACTGCAG	*scyA*-14-*lacZ* fusion in pKM232
scyA_R25	CCCCAAGCTTTTCAGCATCTTGAGCTGAAAC	*scyA*-25-*lacZ* fusion in pKM232
cctA_F(+1)	CGGGGTACCCATAAGTTTGGGTTGCTTATTAC	*cctA*-*lacZ* fusion in pKM232
cctA_R	CCCCAAGCTTCGCGGCAAATGCGGTTGG	*cctA*-*lacZ* fusion in pKM232

htpH_F(+1)	CGGGGTACCACTGAGCGTTTGCTTTAACC	*htpG-lacZ* fusion in pKM232
htpG_R	CCCCAAGCTTCTGTTTGACTTCAGTTTGAAA	*htpG-lacZ* fusion in pKM232
SO_0827_F(+1)	CGGGGTACCGTTGCATACACAATACTGTTTAT	*SO_0827-lacZ* fusion in pKM232
SO_0827_R	CCCCAAGCTTAATAGGCAGTAAAGCCACG	*SO_0827-lacZ* fusion in pKM232

[a]Restriction sites used for cloning are underlined
