# Supplementary material for: The Small RNA RyhB Is a Regulator of Cytochrome Expression in Shewanella oneidensis
Source: Front Microbiol. 2018 Feb 21;9:268. doi: 10.3389/fmicb.2018.00268 (PMC5826389; doi:10.3389/fmicb.2018.00268)
Supplement: Supplementary file 3 [file Table3.pdf]

Supplementary Material

The small RNA RyhB is a regulator of cytochrome expression in *Shewanella oneidensis*

Karin L. Meibom*, **Elena M. Cabello, Rizlan Bernier-Latmani**

*** Correspondence:** Karin L. Meibom: karin.meibom@epfl.ch

Supplementary Table 3. Genes expressed at higher level in the Δ*fur* mutant relative to the wild-type strain with a $log_2R>1$ and adjusted p-values <0.05.

Locus	Annotation	log_2R	adj.P.Val
SO_0139	Ferritin Ftn	1.09	1.40E-04
SO_0154	Inner membrane protein of unknown function DUF893	1.33	1.44E-06
SO_0291	Predicted periplasmic protein	1.09	1.61E-05
SO_0296	10 TMS drug/metabolite efflux pump (DME) family	1.84	8.71E-07
SO_0313	Putrescine:ornithine antiporter PotE	1.00	1.06E-02
SO_0334	Protein of unknown function	1.49	5.06E-05
SO_0447	Iron-regulated inner membrane protein	1.40	1.47E-05
SO_0448	Iron-regulated inner membrane protein	1.47	9.45E-06
SO_0449	Iron-regulated inner membrane protein	1.80	6.34E-08
SO_0458	Hypothetical protein	2.55	1.30E-05
SO_0541	RNA-metabolizing metallo-beta-lactamase family protein	1.05	1.21E-05
SO_0552	Predicted periplasmic protein	1.84	6.71E-06
SO_0583	Bacterioferritin-associated ferredoxin Bfd	1.65	4.81E-04
SO_0643	Mu phage transposase OrfA TnpA_MuSo1a	1.27	3.42E-03
SO_0644	Mu phage transposase OrfB TnpA_MuSo1b	2.72	2.25E-04
SO_0645	Mu phage uncharacterized protein	2.19	3.67E-04
SO_0648	Mu phage protein of unknown function	2.21	9.18E-05

SO_0649	Mu phage uncharacterized protein	2.29	4.57E-05
SO_0674	Mu phage peptidase	3.62	4.71E-05
SO_0675	Mu phage major head subunit	4.58	9.29E-06
SO_0690	Mu phage DNA adenine methylase Dam family	2.00	3.10E-04
SO_0703	Chaperonin GroES	1.04	3.56E-02
SO_0704	Chaperonin GroEL	1.27	1.56E-02
SO_0715	Sulfite dehydrogenase molybdopterin-binding subunit SorA	3.13	8.62E-05
SO_0725	Bifunctional catalase/peroxidase HPI KatG	1.03	2.83E-04
SO_0742	ABC-type Fe3+ uptake system ATPase component FbpC	1.33	1.83E-05
SO_0743	ABC-type Fe3+ uptake system permease component FbpB	1.06	1.98E-04
SO_0744	ABC-type Fe3+ uptake system substrate-binding component FbpA	1.45	2.75E-05
SO_0748	Hypothetical protein	1.36	3.34E-05
SO_0762	Isochorismate hydrolase family protein	1.45	2.30E-05
SO_0797	Periplasmic thioredoxin-family protein	3.50	1.62E-06
SO_0798	TonB-dependent receptor	4.37	3.98E-09
SO_0799	Protein of unknown function	1.34	1.61E-05
SO_0809	Periplasmic azurin Azu	3.04	7.78E-07
SO_0820	ABC-type macrolide export systemMFP component MacA	1.12	3.12E-05
SO_0855	Hydroxyneurosporene synthase AttH	1.06	4.34E-06
SO_0856	ABC-type export system permease component AttFG	1.01	1.26E-05
SO_0857	ABC-type export system ATPase component AttE	1.19	2.54E-05
SO_0860	Two component signal transduction system response regulator with HD domain	1.05	3.89E-05
SO_0895	Pirin family protein	1.32	2.16E-06
SO_0916	Bifunctional MarR family transcriptional regulator/acteyltransferase GNAT family	1.39	8.88E-08
SO_0956	Alkyl hydroperoxide reductase flavoprotein component AhpF	1.12	4.15E-02
SO_0958	Alkyl hydroperoxide reductase peroxiredoxin component AhpC	1.45	6.52E-04
SO_1114	DNA polymerase IV	1.78	2.24E-08
SO_1188	Inner membrane protein with PepSY TM helix	2.00	4.21E-06
SO_1189	Putative periplasmic protein of unknown function	2.31	1.30E-09
SO_1190	Putative periplasmic CbiK superfamily protein	2.48	2.05E-09
SO_1245	Putative arginine uptake porter ArgW	1.32	7.52E-05
SO_1397	Cytosine deaminase	1.25	6.36E-06
SO_1482	TonB-dependent receptor	5.22	1.27E-08
SO_1483	Malate synthase AceB	2.65	1.16E-08
SO_1484	Isocitrate lyase AceA	2.68	2.43E-07
SO_1532	Predicted periplasmic protein	1.55	3.32E-04

SO_1571	Bifunctional DNA-binding protein / oxidoreductase	1.23	1.02E-04
SO_1576	GSH-dependent disulfide-bond oxidoreductase	1.00	7.02E-07
SO_1580	Hemoglobin/transferrin/lactoferrin receptor protein	4.15	3.44E-10
SO_1655	3'(2'),5'-bisphosphate nucleotidase CysQ	1.15	8.21E-04
SO_1656	D-hexose 6-phosphotransferase HexA	1.02	1.16E-03
SO_1666	Phenylalanine-4-hydroxylase PhhA	1.23	2.64E-05
SO_1667	4a-hydroxytetrahydrobiopterin dehydratase PhhB	1.08	9.29E-06
SO_1698	Autocatalytic aspartic peptidase	1.21	9.94E-05
SO_1699	Transmembrane transcriptional regulator	1.47	2.86E-06
SO_1700	Putative lipoprotein	1.13	3.31E-04
SO_1751	ABC-type transport system bifunctional permease and peptidase M1 family component	1.03	1.53E-04
SO_1755	phosphoglucomutase/phosphomannomutase family protein	2.23	7.13E-09
SO_1756	Glyoxalase family protein	2.45	1.10E-07
SO_1757	Transcription activator-like effector binding domain protein	2.26	5.55E-07
SO_1758	Transcriptional repressor DeoR family	1.44	1.76E-06
SO_1784	Ferrous iron transport protein B FeoB	1.19	3.77E-07
SO_1787	Inner membrane protein of unknown function	1.26	3.72E-04
SO_1855	Ribosome modulation factor	2.70	3.69E-09
SO_1881	Membrane fusion protein, multidrug efflux system	2.23	6.21E-09
SO_1882	K18138 multidrug efflux pump	1.64	2.78E-07
SO_1962	4-hydroxyphenylpyruvate dioxygenase HppD	2.30	3.18E-05
SO_1963	Homogentisate 1,2-dioxygenase HmgA	2.21	9.45E-06
SO_1967	Predicted membrane protein	1.31	1.14E-07
SO_2016	Heat shock chaperone HtpG	1.06	4.86E-02
SO_2017	Heat shock response protein	1.38	1.56E-02
SO_2045	Cation efflux protein CDF family	1.15	6.00E-06
SO_2228	CBS domain containing protein	1.21	4.05E-06
SO_2280	Drug:H+ antiporter DHA1 family	1.69	7.56E-08
SO_2367	Pseudogene	2.46	2.40E-07
SO_2426	Two-component system, OmpR family, response regulator	2.96	7.56E-08
SO_2664	Mu phage uncharacterized protein	1.13	4.15E-03
SO_2666	Mu phage uncharacterized protein	1.19	1.40E-03
SO_2667	Mu phage host gene modulation protein GemA	1.28	4.83E-04
SO_2768	Acyl-CoA dehydrogenase	1.52	1.38E-05
SO_2841	Hypothetical protein	2.09	8.71E-07
SO_2882	Serine protein kinase PrkA	1.27	3.56E-06
SO_2883	Protein of unknown function	1.09	1.37E-04

SO_2884	SpoVR family protein	1.33	3.24E-06
SO_2893	Base-induced periplasmic protein YceI	1.08	5.22E-06
SO_2939	Lambda phage uncharacterized protein	2.31	1.49E-05
SO_2940	Lambda phage tail fiber protein J	3.82	1.05E-06
SO_2941	Lambda phage tail assembly protein I	4.99	3.41E-06
SO_2943	Pseudogene	2.52	3.41E-05
SO_2944	Lambda phage structural protein	3.41	1.05E-06
SO_2945	Lambda phage tail fiber protein	4.66	4.26E-07
SO_2946	Lambda phage protein with carbohydrate-binding module	4.75	3.88E-07
SO_2947	Lambda phage protein of known function	4.71	1.71E-06
SO_2948	Lambda phage tail assembly protein K	2.84	2.36E-05
SO_2949	Lambda phage minor tail protein L	2.19	1.02E-05
SO_2950	Lambda phage uncharacterized protein	2.23	1.04E-05
SO_2951	Lambda phage protein of known function	2.56	3.33E-05
SO_2953	Lambda phage tail length tape meausure protein H	4.49	2.46E-06
SO_2954	Lambda phage uncharacterized protein	4.81	1.50E-06
SO_2955	Lambda phage minor tail protein G	4.69	6.21E-06
SO_2956	Lambda phage major tail protein V	4.55	1.11E-06
SO_2957	Lambda phage protein of unknown function	4.41	1.35E-05
SO_2958	Lambda phage protein HK97-gp10 family	4.39	7.94E-06
SO_2960	Lambda phage phage head-tail joining protein	4.21	6.19E-06
SO_2961	Lambda phage head-tail connector protein HK97-gp6 family	4.26	5.71E-06
SO_2962	Lambda phage helical domain protein	4.51	7.02E-07
SO_2963	Lambda phage major capsid protein	4.82	1.29E-06
SO_2964	Lambda phage head maturation protease	5.04	1.16E-06
SO_2965	Lambda phage portal protein B	4.59	3.78E-06
SO_2968	Lambda phage terminase A	4.77	1.56E-05
SO_2969	Lambda phage endonuclease HNH family	4.44	9.87E-06
SO_2970	Lambda phage uncharacterized protein	4.39	2.04E-05
SO_2971	Lambda phage holin S	4.59	1.07E-05
SO_2972	Lambda phage uncharacterized protein	4.33	2.77E-05
SO_2973	Lambda phage lysozyme R	4.53	1.97E-05
SO_2974	Lambda phage pyridoxal phosphate dependent enzyme	4.44	3.19E-05
SO_2976	Lambda phage uncharacterized protein	1.66	2.44E-04
SO_2977	Lambda phage uncharacterized protein	1.51	1.48E-03
SO_2978	Lambda phage integrase	2.25	6.01E-05
SO_2979	Lambda phage uncharacterized protein	2.51	9.57E-06
SO_2981	Lambda phage uncharacterized protein	1.96	3.33E-04

SO_2982	Lambda phage uncharacterized protein	1.74	4.99E-04
SO_2983	Lambda phage uncharacterized protein	2.31	1.48E-04
SO_2984	Lambda phage replication protein P	1.89	1.77E-04
SO_2985	Lambda phage replication protein O	1.50	5.53E-04
SO_2986	Lambda phage uncharacterized protein	1.82	6.99E-04
SO_2987	Lambda phage uncharacterized protein	1.76	1.81E-03
SO_2988	Lambda phage phage regulatory protein CII	2.40	4.84E-05
SO_2989	Lambda phage transcriptional repressor of early genes Cro	2.33	9.17E-05
SO_2993	Lambda phage type II DNA modification methyltransferase	2.20	1.80E-04
SO_2997	Lambda phage uncharacterized protein	1.22	4.85E-03
SO_3000	Lambda phage uncharacterized protein	1.55	1.02E-04
SO_3001	Lambda phage uncharacterized protein	1.62	1.67E-04
SO_3002	Lambda phage uncharacterized protein	1.62	2.14E-04
SO_3003	Lambda phage uncharacterized protein	1.48	1.24E-04
SO_3004	Lambda phage type II restriction-modification system DNA N-6-adenine-methyltransferase Dam	1.81	3.99E-05
SO_3005	Lambda phage uncharacterized protein	1.61	3.05E-04
SO_3006	Lambda phage type II restriction modification system cytosine-5 DNA methyltransferase Dcm	1.72	5.29E-05
SO_3010	Lambda phage uncharacterized protein	1.23	7.90E-03
SO_3012	Lambda phage DNA excisionase Xis	1.33	1.66E-04
SO_3025	Periplasmic siderophore cleavage esterase IroE family	2.90	1.30E-09
SO_3030	Putrescine monooxygenase PubA	6.39	3.14E-09
SO_3031	N-hydroxyputrescine-succinyl CoA transferase PubB	6.33	6.20E-10
SO_3032	NTP-dependent putrebactin synthetase PubC	6.20	3.47E-10
SO_3033	TonB-dependent ferric putrebactin siderophore receptor PutA	6.12	3.44E-10
SO_3034	Ferric putrebactin reductase PutB	6.08	9.47E-10
SO_3061	DNA topoisomerase III TopB	1.40	1.35E-05
SO_3062	Predicted inner membrane protein	5.86	3.47E-10
SO_3063	Alanine or glycine:cation symporter, AGCS family	4.35	2.42E-10
SO_3087	Predicted membrane protein	1.19	1.12E-05
SO_3108	Bifunctional 13-dimethyluroporphyrinogen III dehydrogenase/siroheme ferrochelatase	1.05	1.88E-03
SO_3119	Hypothetical protein	1.04	1.30E-05
SO_3333	Periplasmic substrate binding protein family 1	1.07	2.12E-06
SO_3343	Predicted lipoprotein	1.08	1.44E-06
SO_3344	Predicted inner membrane protein	5.14	8.71E-07
SO_3370	UPF0312 family alkali-inducible periplasmic protein YceI	2.64	3.59E-06

SO_3371	Cytochrome B561 YceJ	2.41	4.10E-05
SO_3373	Hypothetical protein	1.52	2.77E-05
SO_3392	NAD(P)H:flavin oxidoreductase Sye4	1.39	5.14E-03
SO_3406	Putative siderophore transporter component 1	2.68	1.20E-06
SO_3407	Putative siderophore transporter component 2	3.10	3.35E-07
SO_3408	Putative siderophore transporter component 3	3.06	6.20E-10
SO_3430	Recombination protein RecA	1.34	5.44E-08
SO_3462	DNA repair protein RecN	1.91	3.97E-07
SO_3577	Stress-induced multi-chaperone system component ClpB	1.17	1.27E-02
SO_3583	16S rRNA pseudouridine516 synthase	2.95	1.30E-09
SO_3584	Superoxide-responsive transcriptional repressor of superoxide stress response AzrR	3.00	7.63E-10
SO_3585	NADPH-dependent azoreductase Azr	6.61	6.21E-09
SO_3586	Glyoxalase family protein	5.96	1.30E-09
SO_3587	Hypothetical protein	4.79	6.67E-10
SO_3665	ABC-type multidrug eflux system bifunctional ATPase and permease component	2.42	4.19E-08
SO_3667	Heme iron utilization protein HmuZ	6.23	1.77E-08
SO_3668	Heme iron utilization protein HmuX	6.21	1.30E-09
SO_3669	Hemoglobin/transferrin/lactoferrin receptor protein HmuA	6.28	2.66E-08
SO_3670	Periplasmic protein TonB	5.41	1.14E-07
SO_3671	TonB1 energy transduction system for heme uptake inner membrane component ExbB	6.00	7.56E-08
SO_3672	TonB1 energy transduction system for heme uptake inner membrane component ExbD	5.85	4.09E-08
SO_3673	Iron complex transport system substrate-binding protein HmuB	6.15	1.01E-08
SO_3674	Iron complex transport system permease protein HmuC	5.46	3.67E-10
SO_3675	Iron complex transport system ATP-binding protein HmuD	5.98	3.44E-10
SO_3676	Protein of unknown function	3.01	3.44E-10
SO_3716	DnaJ domain protein	1.02	1.98E-05
SO_3723	Adenylyl-sulfate kinase	1.71	4.20E-02
SO_3724	Pseudogene	1.63	4.58E-02
SO_3726	Sulfate adenylyltransferase large subunit CysN	1.59	4.13E-02
SO_3727	Sulfate adenylyltransferase small subunit CysD	1.68	2.92E-02
SO_3728	Uroporphyrin-III C-methyltransferase CysG	1.78	3.78E-02
SO_3888	Uncharacterized protein	1.67	2.60E-08
SO_3913	Fur regulated PKHD-type hydroxylase PiuC	3.92	6.21E-09
SO_3914	TonB dependent receptor	4.79	1.01E-08

SO_3915	cheX; chemotaxis signal transduction system CheY dephosphorylase CheX	1.46	2.38E-06
SO_4303	ATPase family protein associated with various cellular activities	1.15	9.34E-05
SO_4317	Biofilm-promoting protein BpfA	1.01	2.96E-05
SO_4396	FMN-dependent NADH-azoreductase AzoR	1.22	1.87E-06
SO_4446	ABC-type molybdate uptake system ATPase component ModC	1.01	1.26E-05
SO_4474	Pseudogene	1.12	2.38E-06
SO_4477	Two-component system, OmpR family, response regulator CpxR	1.29	9.87E-08
SO_4478	Two-component system, OmpR family, sensor histidine kinase CpxA	1.20	1.54E-07
SO_4480	NAD+-dependent acetaldehyde dehydrogenase ExaC	1.81	7.12E-06
SO_4492	Protein of unknown function	1.23	1.62E-02
SO_4516	TonB-dependent siderophore receptor	5.20	3.44E-10
SO_4523	Iron-responsive TonB-dependent enterobactin receptor IrgA	5.57	2.14E-07
SO_4524	Transcriptional regulator LysR family	2.76	8.71E-07
SO_4558	Hypothetical protein	2.14	6.67E-10
SO_4564	TonB mediated energy transduction system energy transducer component TonB	1.49	1.40E-05
SO_4603	Bifunctional transcriptional repressor of SOS-response/self-cleaving protease LexA	1.86	2.33E-07
SO_4604	Cell division inhibitor SulA	1.92	2.97E-07
SO_4606	aa3-type cytochrome c oxidase subunit II CoxB	1.35	1.68E-05
SO_4627	Glutamine rich protein	1.96	4.47E-08
SO_4650	Alpha/beta hydrolase family of unknown function	2.52	9.87E-03
SO_4651	Protein of unknown function	2.11	1.47E-02
SO_4652	ABC-type sulfate/thiosulfate uptakesystem substrate-binding subunit Sbp	3.02	4.86E-03
SO_4653	ABC-type sulfate/thiosulfate uptakesystem permease component 1 CysT	2.95	8.02E-03
SO_4654	ABC-type sulfate/thiosulfate uptakesystem permease component 2 CysW	2.39	1.62E-02
SO_4655	ABC-type sulfate/thiosulfate uptakesystem ATPase component CysA	2.33	1.99E-02
SO_4664	Predicted membrane protein	1.05	2.79E-04
SO_4708	Putative sulfate exporter (PSE) family protein	1.37	2.64E-02
SO_4740	Predicted membrane protein of unknown function	3.94	3.77E-07
SO_4743	Iron complex outermembrane recepter protein	1.89	1.92E-05
SO_4782	Protein with c-terminal DUF1078 domain	1.53	5.32E-08
SO_4787	Mu phage uncharacterized protein	1.12	9.25E-03
SO_4788	Mu phage uncharacterized protein	1.22	7.70E-03

SO_4790	Lambda phage uncharacterized protein	2.43	1.18E-05
SO_4793	Lambda phage uncharacterized protein	2.90	1.97E-04
SO_4795	Lambda phage uncharacterized protein	1.61	1.66E-04
SO_4819	Hypothetical protein	3.53	1.80E-07
SO_A0012	SOS mutagenesis protein RulB	1.71	3.33E-05
SO_A0013	SOS mutagenesis protein RulA	1.91	2.07E-05
SO_A0047	Hypothetical protein	1.57	4.10E-05
SO_A0049	Toxin secretion ABC transporter, ATP-binding subunit/permease protein, putative	1.11	1.54E-04
SO_A0099	Hypothetical protein	1.28	3.97E-07
SO_A0176	Hypothetical protein	1.48	2.36E-03
SO_A0177	Bacteriocin-like peptide	2.00	1.36E-05
SO_A0178	Bacteriocin-like peptide	2.26	6.68E-06
SO_A0181	Pseudogene	1.20	4.12E-05
SO_m007	RyhB	1.35	2.01E-03
