# Supplementary material for: The Small RNA RyhB Is a Regulator of Cytochrome Expression in Shewanella oneidensis
Source: Front Microbiol. 2018 Feb 21;9:268. doi: 10.3389/fmicb.2018.00268 (PMC5826389; doi:10.3389/fmicb.2018.00268)
Supplement: Supplementary file 4 [file DataSheet1.PDF]

Supplementary Material

The small RNA RyhB is a regulator of cytochrome expression in *Shewanella oneidensis*

Karin L. Meibom*, Elena M. Cabello, Rizlan Bernier-Latmani

*** Correspondence:** Karin L. Meibom: karin.meibom@epfl.ch

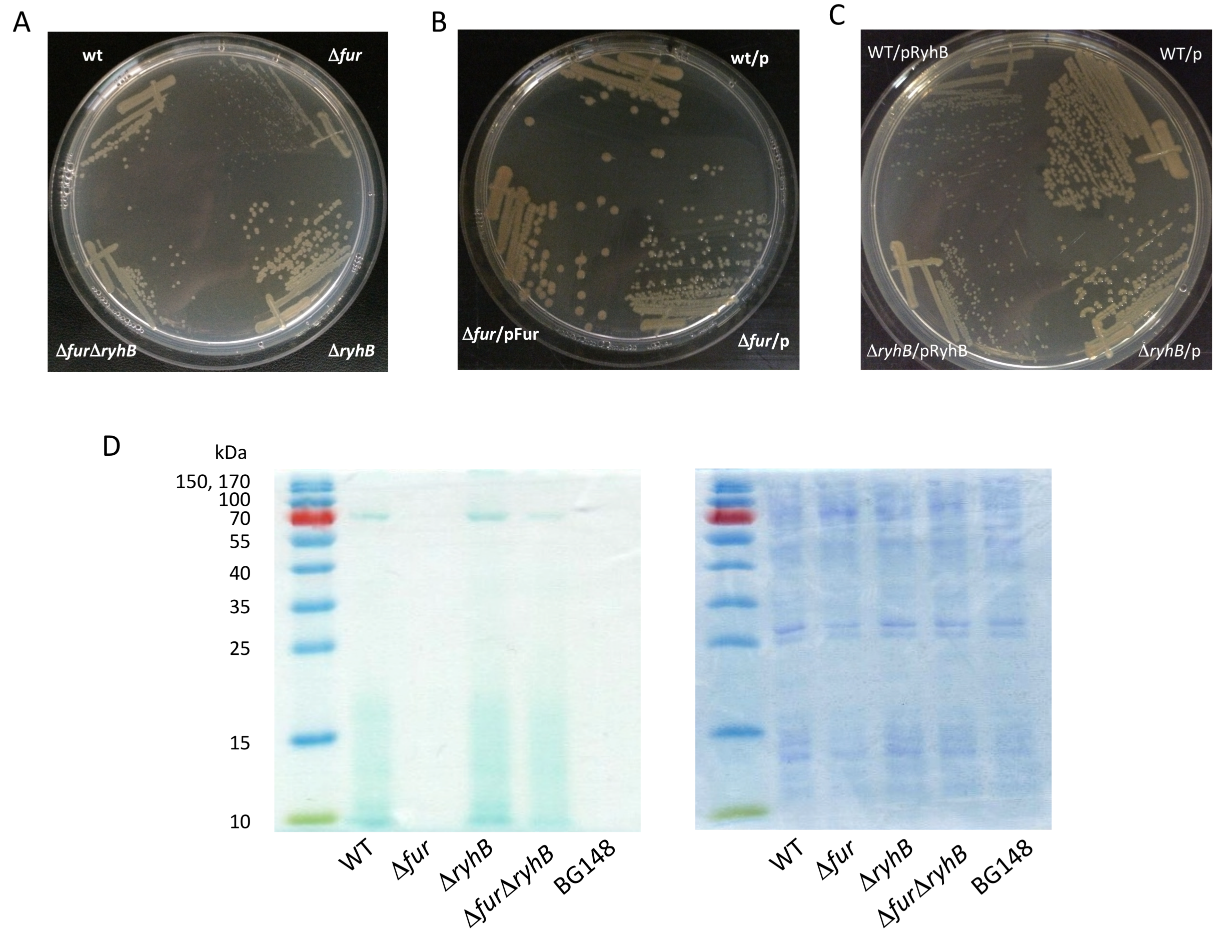

Supplementary Figure 1. Fur and RyhB influences growth and heme production. (A) Colonies of *S. oneidensis* wild-type, Δ*fur*, Δ*ryhB*, and Δ*furΔryhB* strains grown under aerobic conditions on LB agar plates at 30°C. (B) Colonies formed by wild-type and the Δ*fur* mutant containing an empty plasmid pHGE-P$_{tac}$ (designated p) and Δ*fur* containing pHGE-P$_{tac}$-fur (designated pFur) on LB agar plates containing IPTG to induce expression of *fur*. (C) *S. oneidensis* wild-type and Δ*ryhB* strains containing an empty plasmid (pKM033; p) or plasmid expressing RyhB (pKM033-ryhB; pRyhB) on LB agar plates with IPTG to induce expression of *ryhB*. (D) Bacterial lysates (10µg total protein) were separated on a 15% SDS polyacrylamide gel and then gel heme-stained (Thomas et al., 1976) (left panel) or stained with Coomassie Blue (right panel). The strain BG148 that contains a transposon insertion in *ccmC* gene (a cytochrome *c* maturation gene) (Bouhenni et al., 2005) was used as a control.

Bouhenni, R., Gehrke, A., and Saffarini, D. (2005). Identification of genes involved in cytochrome c biogenesis in Shewanella oneidensis, using a modified mariner transposon. Appl. Environ. Microbiol. *71*, 4935–4937.

Thomas, P.E., Ryan, D., and Levin, W. (1976). An improved staining procedure for the detection of the peroxidase activity of cytochrome P-450 on sodium dodecyl sulfate polyacrylamide gels. Anal. Biochem. *75*, 168–176.
