# Supplementary material for: The Small RNA RyhB Is a Regulator of Cytochrome Expression in Shewanella oneidensis
Source: Front Microbiol. 2018 Feb 21;9:268. doi: 10.3389/fmicb.2018.00268 (PMC5826389; doi:10.3389/fmicb.2018.00268)
Supplement: Supplementary file 5 [file DataSheet2.PDF]

Supplementary Material

The small RNA RyhB is a regulator of cytochrome expression in *Shewanella oneidensis*

Karin L. Meibom[*], Elena M. Cabello, Rizlan Bernier-Latmani

*** Correspondence:** Karin L. Meibom: karin.meibom@epfl.ch

A

SO_4716->ACGCGCCATCAATAAAAATGTGCATATTTGCGAGAAATAGCTTGCAAAGCTA
-35

Fur-box +1 RyhB
ATT**GAGAATGATTATCGTTAAC**TTGCGTTCCAAAACTCATCTTTAACTCTGATGACTGGTAA
-10

TCTGACGTTGTTAAAGTGCTCCTGAGTTTGCAAGCACGACATTGCTCACACACTCTTTTGTG

Poly(U)tail
GCCGGATTCATCATCCGGCTTTTTTTTGCCTGTTATTCAGCGAATAGGCGCATTAGGCTAAC
rho-independent terminator

CATGTCTAGGTTTGCGCCACAT<-SO_4717

B

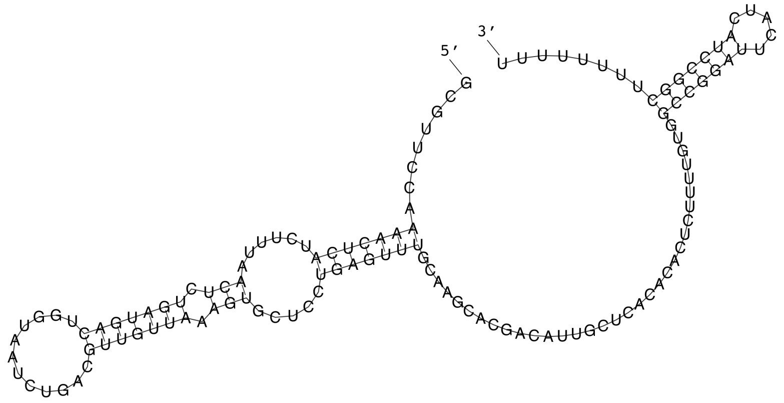

C

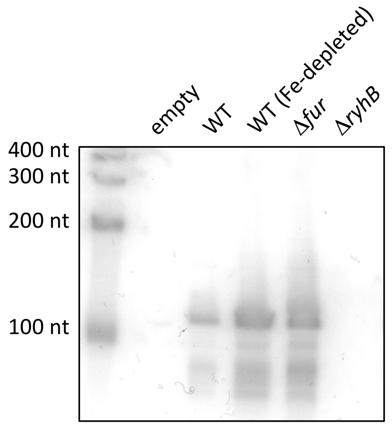

Supplementary Figure 2. Genomic location, predicted secondary structure and expression of RyhB. (A) Nucleotide sequence of the intergenic region containing *ryhB* between SO_4716 and SO_4717. The putative binding site for Fur (Fur box) is marked in bold, the putative -10 and -35 sequences are underlined. The transcription start site (Shao et al., 2014) is indicated by an arrow and the assumed transcribed sequence presented in red. The rho-independent terminator was predicted by ARNold and is underlined (Naville et al., 2011) (http://rna.igmors.u-psud.fr/toolbox/arnold/). (B) RyhB seconday structure as predicted by RNAfold (http://rna.tbi.univie.ac.at/cgi-bin/RNAWebSuite/RNAfold.cgi). (C) Northern blot probing RyhB. 10 µg of total RNA from indicated *S. oneidensis* strains was migrated on a 8M urea / 6% polyacrylamide gel and electroblotted onto a Amersham Hybond-N+ membrane. Transferred RNA were cross-linked to the membrane with a Stratagen UV cross-linker and subsequently hybridized overnight with a 5'-biotinylated oligonucleotide probe at 42 °C using UltraHybOligo hybridization buffer (Ambion). The North2South chemiluminescent detection kit (Thermo Scientific) was used for signal detection. The Δ*ryhB* strain was used as a negative control. The left lane shows a RNA marker (RNA Century Marker, Ambion).

Naville, M., Ghuillot-Gaudeffroy, A., Marchais, A., and Gautheret, D. (2011). ARNold: a web tool for the prediction of Rho-independent transcription terminators. RNA Biol. *8*, 11–13.

Shao, W., Price, M.N., Deutschbauer, A.M., Romine, M.F., and Arkin, A.P. (2014). Conservation of Transcription Start Sites within Genes across a Bacterial Genus. MBio *5*, e01398-14-e01398-14.
