# Supplementary material for: The Small RNA RyhB Is a Regulator of Cytochrome Expression in Shewanella oneidensis
Source: Front Microbiol. 2018 Feb 21;9:268. doi: 10.3389/fmicb.2018.00268 (PMC5826389; doi:10.3389/fmicb.2018.00268)
Supplement: Supplementary file 6 [file DataSheet3.PDF]

Supplementary Material

The small RNA RyhB is a regulator of cytochrome expression in *Shewanella oneidensis*

Karin L. Meibom*, Elena M. Cabello, Rizlan Bernier-Latmani

*** Correspondence:** Karin L. Meibom: karin.meibom@epfl.ch

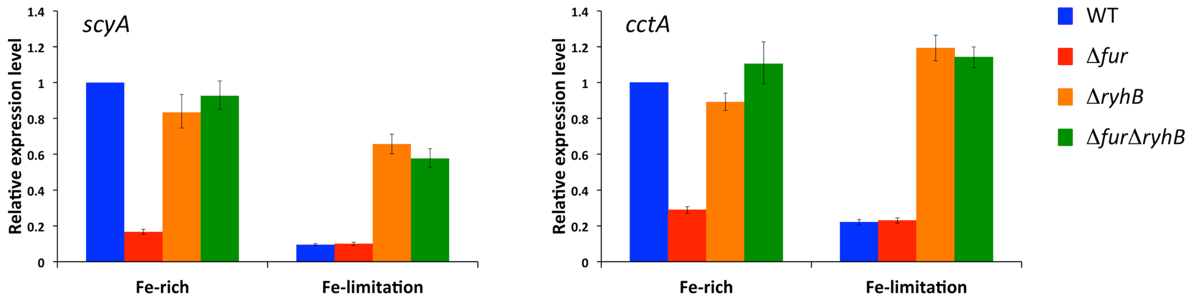

Supplementary Figure 3. Expression of *scyA* and *cctA* is regulated by iron-availability through Fur and RyhB. Transcript levels were assessed by qRT-PCR in *S. oneidensis* wild-type, Δ*fur*, Δ*ryhB*, and *Δfur*Δ*ryhB* strains in rich medium (iron-rich) and after 30 min growth with iron chelator 2,2'-dipyridyl (250µM) (iron-limitation). Transcript levels are shown relative to wild-type level in rich medium as expression ratio of four technical replicates (error bars, standard error). Results shown are from an independent experiment to the data presented in Figure 2.
