# Supplementary material for: The Small RNA RyhB Is a Regulator of Cytochrome Expression in Shewanella oneidensis
Source: Front Microbiol. 2018 Feb 21;9:268. doi: 10.3389/fmicb.2018.00268 (PMC5826389; doi:10.3389/fmicb.2018.00268)
Supplement: Supplementary file 7 [file Image4.pdf]

# *Supplementary Material*

# The small RNA RyhB is a regulator of cytochrome expression in *Shewanella oneidensis*

**Karin L. Meibom***, **Elena M. Cabello, Rizlan Bernier-Latmani**

*** Correspondence:** Karin L. Meibom: karin.meibom@epfl.ch

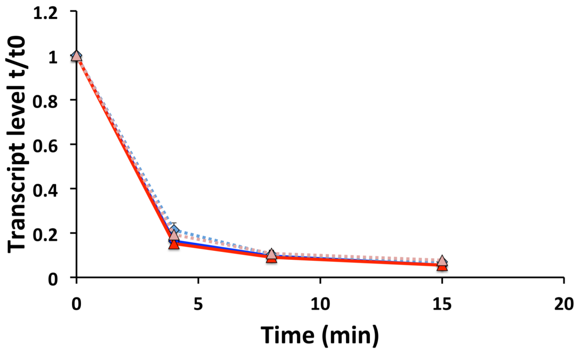


**Supplementary Figure 4.** Production of RyhB does not affect the stability of all mRNAs. Transcript levels of the *recA* gene at 0, 4, 8, and 15 min after transcription was stopped in the Δ*ryhB* mutant containing an empty plasmid (pKM033; p, blue diamonds) or a plasmid expressing RyhB (pKM033-ryhB; pRyhB, red triangles). qRT-PCR was used to determine the transcript level relative to time 0, just before addition of rifampicin. Data are from two individual experiments; with four replicate cDNA samples used in qPCR and shown as expression ratio ± standard error. Results shown in dark colors and solid lines are from one experiment and in light colors and dashed lines from a second experiment.
