# Supplementary material for: The Small RNA RyhB Is a Regulator of Cytochrome Expression in Shewanella oneidensis
Source: Front Microbiol. 2018 Feb 21;9:268. doi: 10.3389/fmicb.2018.00268 (PMC5826389; doi:10.3389/fmicb.2018.00268)
Supplement: Supplementary file 8 [file DataSheet5.pdf]

frontiers

# *Supplementary Material*

# The small RNA RyhB is a regulator of cytochrome expression in *Shewanella oneidensis*

**Karin L. Meibom***, **Elena M. Cabello, Rizlan Bernier-Latmani**

*** Correspondence:** Karin L. Meibom: karin.meibom@epfl.ch

## SUPPLEMENTARY MATERIALS AND METHODS

**Construction of pHGE-P*tac-fur*.** The complementing plasmid expressing Fur from the IPTG-inducible promoter P*tac* was constructed by amplifying *fur* (using primers fur_start and fur_stop) and cloning into the *Eco*RI site of plasmid pHGE-P*tac* (Luo et al., 2013). Correct orientation of the gene was verified by sequencing.

**Heme staining.** Bacterial strains were grown to early stationary phase ($OD_{600nm}$ 1.8-2.0) and 10 ml of each culture was harvested and the bacterial pellet washed once with PBS. Cells were lysed in PBS using a Precellys 24 homogenizer (microorganism lysing kit VK01) with three cycles of 30 s at 6400 rpm with 5 min breaks on ice between cycles. Total protein concentrations in the bacterial lysates were determined using a BCA protein assay (Pierce Chemical). 10 µg of proteins were resolved by SDS-PAGE gel using 15% polyacrylamide gels and stained with 3,3',5,5'-tetramethylbenzidine as described (Thomas et al., 1976).

**Northern blot.** 10 µg of total RNA from indicated *S. oneidensis* strains was migrated on a 8M urea / 6% polyacrylamide gel and electroblotted onto a Amersham Hybond-N+ membrane. Transferred RNA were cross-linked to the membrane with a Stratagen UV cross-linker and subsequently hybridized overnight with a 5'-biotinylated oligonucleotide probe at 42 °C using UltraHybOligo hybridization buffer (Ambion). The North2South chemiluminescent detection kit (Thermo Scientific) was used for signal detection. The Δ*ryhB* strain was used as a negative control. The left lane shows a RNA marker (RNA Century Marker, Ambion).

Luo, Q., Dong, Y., Chen, H., and Gao, H. (2013). Mislocalization of Rieske protein PetA predominantly accounts for the aerobic growth defect of Tat mutants in Shewanella oneidensis. PloS One *8*, e62064.

Thomas, P.E., Ryan, D., and Levin, W. (1976). An improved staining procedure for the detection of the peroxidase activity of cytochrome P-450 on sodium dodecyl sulfate polyacrylamide gels. Anal. Biochem. *75*, 168–176.
